# Safe Curing Limits of Thick Composite Shells

**DOI:** 10.3390/ma19143132

**Published:** 2026-07-21

**Authors:** Rikard Gebart

**Affiliations:** Engineering Sciences and Mathematics, Luleå University of Technology, S-97187 Luleå, Sweden; rikard.gebart@associated.ltu.se

**Keywords:** thermal runaway, composite curing, thick composite laminates, shell structures, composite pipes, stability criterion, perturbation analysis, Damköhler number, Biot number, process design

## Abstract

Thermal runaway during cure limits robust process design for thick composite laminates, especially when shell curvature and non-uniform heat transfer alter local heat removal. We present a semi-analytical framework for estimating safe curing limits in curved composite shells by reducing the three-dimensional thermo-kinetic problem to a locally one-dimensional through-thickness stability problem evaluated pointwise over the mid-surface. The resulting criterion is expressed in terms of a critical Damköhler number and separates geometry and boundary heat transfer, represented by a stability factor depending on principal curvatures and Biot numbers, from chemistry and processing temperature, represented by Arrhenius scaling and an effective kinetic factor. The geometry-dependent stability factor is obtained from a nonlinear boundary-value problem and represented by compact differentiable response surfaces for symmetric and asymmetric boundary conditions. Validation against fully coupled transient simulations confirms the predicted separation over the investigated parameter range. A complementary analytical and quasi-three-dimensional flux-ratio assessment shows that lateral heat transport remains small for the representative smooth shell geometries studied, with strongly anticlastic regions providing the most restrictive cases. The framework enables rapid curvature-based stability sweeps, identification of critical locations, estimation of safe thickness limits, and practical screening of cure-cycle modifications without full three-dimensional simulation.

## 1. Introduction

The curing of thick fibre-reinforced polymer laminates (FRP) is challenging due to the strong exothermic nature of thermoset cross-linking together with the low thermal conductivity of polymer composites. When the heat generated by the curing reaction cannot be dissipated efficiently, large temperature gradients can develop within the laminate. These gradients may lead to a heterogeneous degree of cure, accumulation of residual stresses, matrix degradation, or even delamination. During cure, chemical shrinkage and modulus development further induce residual stresses and warpage [1], which in turn can initiate transverse cracking and delamination [2]. Thermal control is therefore essential for both dimensional stability and structural integrity.

Industrial cure cycles are commonly developed on the basis of experience with thin laminates, where heat removal is relatively effective and strong temperature gradients do not arise. However, as laminate thickness increases or when rapid cure cycles are employed, the balance between heat generation and heat dissipation may be disrupted. Under such conditions, manufacturers rely on numerical simulations to predict thermal behaviour during curing. Fully coupled thermal-kinetic models, typically solved using finite-difference, finite-volume, or finite-element methods, can reproduce thermal gradients and overshoot for a wide range of composite systems [3,4,5]. Recent computational advancements have expanded these numerical approaches to include machine learning surrogate models and multi-objective optimization algorithms, which drastically reduce the computational cost of identifying safe process windows for thick laminates [6,7,8]. Although these models are accurate and versatile, they can be time-consuming to apply in practice, be sensitive to the uncertainty of the kinetic parameters, and offer limited physical insight into the stability boundary that separates safe processing from thermal runaway.

Analytical approaches can provide additional insight by revealing the underlying physics and yielding explicit relations between overheating, material parameters, and geometry. However, analytical predictions of thermal runaway in composite curing remain limited. Previous work established a perturbation-based runaway criterion for flat laminates [9], while Farjas et al. [10] obtained analytical critical-thickness conditions for planar geometries with convective boundaries. In parallel, process-oriented studies [8,11] have applied such flat-plate criteria to define safe operating windows in advanced manufacturing strategies.

Despite these advances, existing analytical criteria are fundamentally restricted to planar or effectively one-dimensional geometries and do not account for curvature effects or spatial variations in heat-transfer conditions. In particular, there is currently no analytical framework that (i) generalizes the stability criterion to curved shell geometries and (ii) expresses the critical condition in a form that clearly separates geometric and boundary effects from chemistry and temperature sensitivity. This limitation restricts the direct applicability of analytical methods in practical manufacturing scenarios involving complex three-dimensional composite structures.

In this work, we derive a stability criterion for thermal runaway during the cure of thick composite shells by reducing the full 3D thermo-kinetic problem to a locally one-dimensional through-thickness model evaluated pointwise over the shell surface. This reduction yields a compact semi-analytical critical condition expressed in terms of standard dimensionless groups. In particular, the Damköhler number (Da) represents the balance between heat generation by the chemical reaction and heat removal by conduction, while the Arrhenius number ($A_{r}$) characterizes the temperature sensitivity of the reaction kinetics. Heat exchange at the laminate surfaces is described using Robin (convective) boundary conditions, leading to Biot numbers (Bi) that quantify the relative strength of surface heat transfer compared to internal conduction. Within this framework, geometry and boundary heat transfer enter only through a single stability factor, while chemistry and processing temperature appear through an explicit Arrhenius scaling.

While the governing stability factor is obtained numerically from a non-linear boundary-value problem, the resulting formulation provides an explicit differentiable representation that separates geometry and boundary effects from chemistry and temperature. We validate the resulting criterion against high-precision transient simulations of the fully coupled thermo-kinetic problem. The validity of the locally one-dimensional reduction is assessed separately through an analytical flux-ratio criterion and an independent quasi-three-dimensional flux evaluation, which together show that transverse gradients dominate for the representative smooth shell geometries investigated. Finally, we introduce an objective numerical classification of runaway onset based on temperature derivatives, providing a consistent way to locate the critical threshold in transient simulations and suggesting a route to real-time process monitoring.

The main contribution of this work is therefore a unified semi-analytical stability framework that extends existing flat-laminate criteria to curved shell geometries, while retaining a physically transparent separation between geometry-driven heat-transfer effects and chemistry-driven thermal sensitivity. In addition, the differentiable stability representation makes it possible to formulate a geometry-based flux-ratio check for the locally one-dimensional approximation. Together, these results allow rapid stability assessment across complex structures without resorting to full three-dimensional simulations in routine process screening.

The paper is organised as follows. Section 2 formulates the locally 1D shell model, introduces the perturbation framework, and derives the stability condition and its parameter separation. Section 3 validates the criterion against fully coupled transient simulations and examines the transient behaviour near criticality, including the validity of the locally one-dimensional approximation. Section 4 discusses the implications for process design and the limits of the underlying assumptions. Appendix A, Appendix B and Appendix C provide the perturbation derivation, numerical procedures, and flux-ratio validity analysis, respectively.

## 2. Theory and Numerical Methods

We formulate the governing thermo-kinetic problem for a general thick-walled shell in body-fitted coordinates and derive a thermal-runaway stability criterion. The formulation starts from the full three-dimensional energy balance with standard cure kinetics, which makes the geometric scope explicit: locally, any smooth shell is characterised by its two principal curvatures. We then introduce a controlled reduction to a locally one-dimensional through-thickness model, yielding a conservative stability problem that can be evaluated pointwise over the surface. Details of the perturbation derivation are given in Appendix A; numerical procedures are documented in Appendix B; and the validity of the locally one-dimensional approximation with respect to lateral heat transport is assessed in Appendix C. To improve readability, the main variables and dimensionless parameters are summarised in the Abbreviations table before Appendix A, and their physical meaning is discussed where first introduced.

### 2.1. Problem Formulation

**Figure 1:**
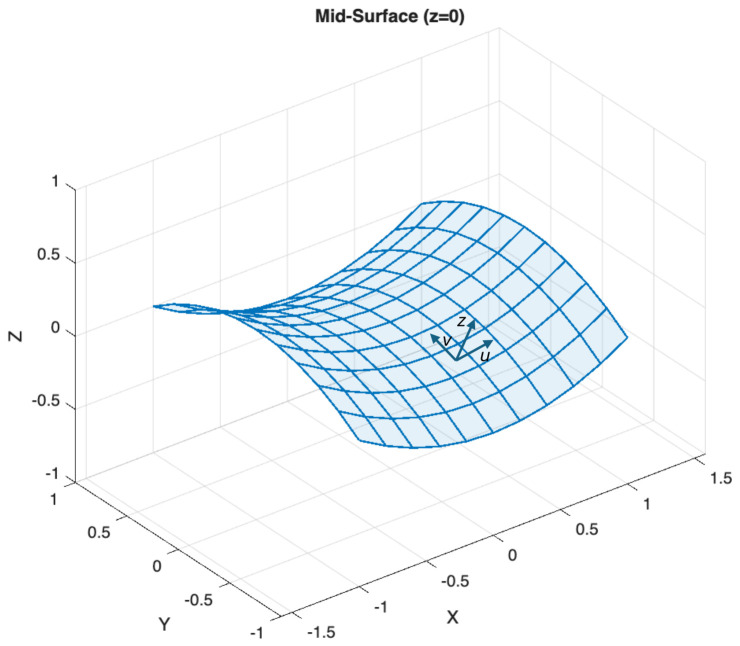
Generic double-curved shell geometry with a body-fitted coordinate system. The in-plane coordinates (u,v) follow the principal curvature directions of the surface, while the transverse coordinate *z* measures the distance through the thickness. This coordinate system allows the three-dimensional problem to be expressed locally as a through-thickness heat-transfer problem.

The theory was developed for a general curved shell of half-thickness *w* whose mid-surface is described by two principal curvatures $\kappa_{1}$ and $\kappa_{2}$. A body-fitted coordinate system (u,v,z) is defined with orthogonal principal-curvature coordinates (u,v) on the mid-surface and a thickness coordinate *z* that measures the distance from the mid-surface along the surface normal (see Figure 1). Any point in the laminate can be expressed as

R(u,v,z)=r(u,v)+zn(u,v),

where z∈[−w,w].

Since the goal is to develop a theory that is valid for general curved shells, the analysis will start from the most general formulation of the energy conservation equation combined with a standard autocatalytic cure kinetic model [12]:

(1) $$\rho c_{p}\frac{\partial T}{\partial t}=\nabla \;\cdot \left(\Lambda \nabla T\right)+\rho q_{c}\frac{\partial \alpha }{\partial t},$$

(2) $$\frac{\partial \alpha }{\partial t}=A\;\exp\;\left(-\frac{E}{RT}\right)\alpha^{m}{\left(1-\alpha \right)}^{n},$$

where *T* is the temperature, α is the degree of cure, Λ is the anisotropic thermal conductivity tensor and all other parameters have their standard meaning in thermo-kinetic modeling and are summarised in the Abbreviations table.

The general problem has no known analytical solution, but it can be reduced to a one-dimensional equation along the thickness direction if lateral conduction along the shell surface is neglected. This approximation decouples neighbouring surface points, so that each point behaves as an independent through-thickness thermal element. For hot regions, lateral conduction would typically redistribute heat toward neighbouring cooler regions and therefore act as a stabilising mechanism. However, the rigorous validity condition is not based on the absolute lateral flux through an element face, but on the net lateral conductive divergence within a local control volume. The magnitude of this omitted term is assessed explicitly in Appendix C.

In a flat laminate, the resulting thermal elements can be viewed as straight columns of constant cross-section. In curved geometries, they deform into frustum-like volumes with thickness-dependent cross-sectional area, reflecting the geometric divergence of the through-thickness heat flux. Once lateral coupling is neglected, each local stability problem depends only on the local principal curvatures and the local boundary heat-transfer conditions. The resulting one-dimensional formulation can therefore be evaluated pointwise over the shell surface.

The validity of this pointwise reduction is governed by the relative magnitude of the omitted lateral conductive divergence compared with the retained through-thickness conductive contribution. The flux-ratio analysis in Appendix C shows that this condition is satisfied for the representative smooth shell geometries investigated here. It also shows that the validity cannot be expressed solely as a bound on curvature magnitude: the controlling quantity is the spatial variation of the geometry-dependent stability factor $\beta_{\mathrm{crit}}$.

In practical terms, the approximation is expected to hold for a wide range of industrial composite structures where heat extraction occurs predominantly through the thickness. However, the model may lose accuracy in situations where lateral heat transport becomes comparable to the transverse flux, such as in regions with strong spatial variations in boundary conditions, highly anisotropic materials with enhanced in-plane conductivity, or localized geometric features that promote lateral heat spreading.

Under this assumption, and using standard differential-geometry identities for orthogonal surface coordinates with principal curvatures $\kappa_{1}$ and $\kappa_{2}$, the energy Equation (1) reduces to

(3) $$\rho c_{p}\frac{\partial T}{\partial t}=\lambda \;\left[\frac{\partial^{2}T}{\partial z^{2}}+\left(\frac{\kappa_{1}}{1+\kappa_{1}z}+\frac{\kappa_{2}}{1+\kappa_{2}z}\right)\frac{\partial T}{\partial z}\right]+\rho q_{c}\frac{\partial \alpha }{\partial t},$$

where λ is the transverse (through-thickness) conductivity associated with Λ. The curvature-dependent terms in Equation (3) arise from the geometric divergence of the heat flux in the thickness direction, reflecting how curvature modifies the local heat-flow pathways. This behaviour admits a simple geometric interpretation: the material can be viewed as a collection of frustum-like through-thickness elements with a cross-sectional area that varies along *z*. The curvature terms in Equation (3) directly account for this variation.

The principal curvatures $\kappa_{1}$ and $\kappa_{2}$ are defined with respect to the surface normal that establishes the thickness coordinate *z*, where one of the two surfaces of the part is chosen to define the positive *z*-direction. This choice is arbitrary and does not affect the physical predictions of the model, but it is required to make the geometry mathematically well defined.

With this convention, a positive curvature corresponds to a surface that bulges in the positive *z*-direction, while a negative curvature corresponds to a surface that curves in the opposite direction. The terms convex and concave are therefore defined relative to this chosen reference orientation.

From a physical perspective, curvature alters the effective cross-sectional area available for through-thickness heat conduction. For a surface that bulges toward the interior of the laminate (i.e., in the direction opposite to the chosen positive *z*-direction), the available conduction area decreases, tending to reduce heat-removal efficiency, whereas curvature in the opposite direction tends to increase it.

Importantly, the net effect of curvature on heat removal depends not only on its sign, but also on the boundary conditions at the two surfaces. In particular, whether a given curvature enhances or reduces heat extraction depends on how the geometry is oriented relative to the more or less effectively cooled surface.

In terms of the dimensionless curvatures $\sigma_{i}=\kappa_{i}w$, this framework provides an intuitive basis for interpreting the role of curvature in the stability maps $\left(\sigma_{1},\sigma_{2}\right)$ presented later.

To accommodate heated tooling, convection heating, or insulating boundaries within a unified mathematical framework, a Robin (convective) boundary condition is applied on both surfaces:

$$-\lambda \frac{\partial T}{\partial n}=h_{\mathrm{eff}}\left(T_{\mathrm{surf}}-T_{\infty }\right),$$

where *n* is the outward unit normal, $h_{\mathrm{eff}}$ $\left[W\;m^{-2}\;K^{-1}\right]$ is the heat transfer coefficient, $T_{\infty }$ [K] is the reference temperature in Newton’s law of cooling [13] and $T_{\mathrm{surf}}$ [K] is the temperature at the surface of the part.

To reveal the governing non-dimensional groups and reduce the total number of independent process parameters, all variables are scaled using the same procedure as in [9]:

- through-thickness coordinate scaled with the laminate half-thickness $w\to \tilde{x}=z/w$,
- time with $\tau =w^{2}\rho c_{p}/\lambda$, which represents the characteristic time scale for heat to diffuse across the laminate thickness $\to \tilde{t}=t/\tau$,
- temperature with the adiabatic temperature rise $\Delta T_{\mathrm{adi}}=q_{c}/c_{p}\to \tilde{T}=T/\Delta T_{\mathrm{adi}}$,
- and curvature with the thickness $w\to \sigma_{i}=\kappa_{i}w$.

With these scalings, the governing equations take the dimensionless form:

(4) $$\frac{\partial \tilde{T}}{\partial \tilde{t}}=\frac{\partial^{2}\tilde{T}}{\partial {\tilde{x}}^{2}}+\left(\frac{\sigma_{1}}{1+\sigma_{1}\tilde{x}}+\frac{\sigma_{2}}{1+\sigma_{2}\tilde{x}}\right)\frac{\partial \tilde{T}}{\partial \tilde{x}}+\frac{\partial \alpha }{\partial \tilde{t}},$$

(5) $$\frac{\partial \alpha }{\partial \tilde{t}}=Da\;\exp\;\left(-\frac{A_{r}}{\tilde{T}}\right)\alpha^{m}{\left(1-\alpha \right)}^{n},$$

with dimensionless boundary conditions

(6) $$\begin{array}{cc} {\left.\frac{\partial \tilde{T}}{\partial \tilde{x}}\right|}_{\tilde{x}=-1}\; & =+\;Bi_{d}\left({\tilde{T}}_{\mathrm{surf},d}-{\tilde{T}}_{\infty }\right), \end{array}$$

(7) $$\begin{array}{cc} {\left.\frac{\partial \tilde{T}}{\partial \tilde{x}}\right|}_{\tilde{x}=+1}\; & =-\;Bi_{u}\left({\tilde{T}}_{\mathrm{surf},u}-{\tilde{T}}_{\infty }\right), \end{array}$$

where the Damköhler number Da, the Arrhenius number $A_{r}$ and the Biot numbers are defined as:

$$Da=\frac{\rho c_{p}Aw^{2}}{\lambda },\;A_{r}=\frac{E}{R\Delta T_{\mathrm{adi}}}\;Bi_{d,u}=\frac{{\left(h_{\mathrm{eff}}\right)}_{d,u}w}{\lambda }.$$

Each of the dimensionless groups has a clear physical interpretation. The Damköhler number Da represents the ratio between heat generation by the chemical reaction and heat removal by conduction. The Arrhenius number $A_{r}$ characterises the thermal sensitivity of the reaction rate, with higher values indicating stronger temperature dependence. The Biot numbers quantify the relative strength of heat transfer at the boundaries compared to internal conduction. For symmetric heating we occasionally shorten the Biot numbers to $Bi_{u}=Bi_{d}=Bi$. Together, these parameters define the balance between heat generation, diffusion, and extraction that governs thermal stability.

In spite of the simplification, the system in Equations (4)–(7) has no known general solution, but it can be solved with the approximate perturbation method discussed in the next section.

### 2.2. Perturbation Solution

From this point onwards, the tilde notation is dropped and all variables are dimensionless unless otherwise stated.

To make the coupled thermal–kinetic problem tractable, the perturbation analysis introduces a controlled sequence of approximations: the kinetic source term is decoupled from the evolving degree of cure, the temperature field is expanded about the reference state, and the resulting stability problem is evaluated in the steady-state limit (see Appendix A).

First, the kinetic term is frozen at its maximum value, replacing

$$f\left(\alpha \right)=\alpha^{m}\;{\left(1-\alpha \right)}^{n}$$

with a constant, $C_{\alpha }=max\left(f\left(\alpha \right)\right)$. This corresponds to the most heat-intensive stage of cure and therefore yields a conservative (worst-case) approximation of the runaway risk. With this approximation, the heat-generation term no longer depends on the evolving degree of cure, which decouples the energy equation from the cure kinetics.

Second, the analysis focuses on the steady-state temperature distribution that would arise if the laminate was exposed to its highest admissible background temperature $T_{\infty }$. Within the decoupled approximation, if the part can reject reaction heat under these stationary worst-case conditions, the preceding transient is not expected to exceed the stability limit. The temperature is therefore written as a small correction to this reference state,

$$T=T_{\infty }+\varepsilon \;\vartheta$$

which leads to a static through-thickness problem for the perturbation temperature profile ϑ(x) (see Appendix A):

(8) $$\vartheta^{\prime \prime }+\left(\frac{\sigma_{1}}{1+\sigma_{1}x}+\frac{\sigma_{2}}{1+\sigma_{2}x}\right)\vartheta^{\prime }+\beta \;e^{\vartheta }=0.$$

Here the dimensionless parameter β

(9) $$\beta =Da\;C_{\alpha }\exp\left(-\frac{A_{r}}{T_{\infty }}\right)\frac{1}{\varepsilon }$$

measures the balance between heat generation and heat removal.

The corresponding boundary conditions involve only the Biot numbers and are independent of the reference temperature $T_{\infty }$, a direct consequence of expanding the temperature field around this reference state:

$$\vartheta^{\prime }\left(-1\right)-Bi_{d}\;\vartheta \left(-1\right)=0,\;\vartheta^{\prime }\left(1\right)+Bi_{u}\;\vartheta \left(1\right)=0.$$

The perturbation problem admits a steady solution only if β is below a certain threshold. When β exceeds this limit, the steady-state temperature profile can no longer exist and the temperature becomes self-accelerating. Because the parameters governing the perturbation equation are the curvatures $\left(\sigma_{1},\sigma_{2}\right)$ and the Biot numbers $\left(Bi_{d},Bi_{u}\right)$, the critical value

$$\beta_{\mathrm{crit}}=\beta_{\mathrm{crit}}\left(\sigma_{1},\sigma_{2},Bi_{d},Bi_{u}\right)$$

is determined entirely by geometry and surface heat transfer.

Once $\beta_{\mathrm{crit}}$ is known, the corresponding critical Damköhler number follows directly from Equation (9):

(10) $$Da_{\mathrm{crit}}=\beta_{\mathrm{crit}}\;\frac{\varepsilon }{C_{\alpha }}\exp\;\left(\frac{A_{r}}{T_{\infty }}\right),$$

This expression separates the effects of geometry and heat transfer (contained in $\beta_{\mathrm{crit}}$) from those of chemistry and processing temperature. The critical Damköhler number can be recast as a criterion for the maximum safe half-thickness of the laminate for given process conditions:

(11) $$w_{\max}=\sqrt{\frac{\lambda }{\rho \;c_{p}}\frac{1}{A}\frac{1}{C_{\alpha }}\;\beta_{\mathrm{crit}}\;\frac{T_{\infty }^{\;2}}{A_{r}}\;\exp\;\left(\frac{A_{r}}{T_{\infty }}\right)}.$$

The perturbation boundary-value problem (BVP), i.e., a differential equation solved subject to boundary conditions at both surfaces, in Equation (8) does not admit a closed-form analytical solution due to its nonlinear structure. However, the problem can be solved numerically with high accuracy for any given combination of curvature and Biot numbers up to the critical limit $\beta_{\mathrm{crit}}$.

A semi-analytical representation is constructed by systematically sampling these numerical solutions over the parameter space and fitting an explicit function to the resulting $\beta_{\mathrm{crit}}$ surface. The determination of $\beta_{\mathrm{crit}}$ for each parameter set is performed through an iterative search for the upper limit of existence of stationary solutions (see Appendix B for details).

Importantly, the shape of the fitted function is not arbitrary, but is constructed in terms of the geometric invariants *S* and *P*, ensuring that it preserves the fundamental symmetries of the exact solution. This ensures that the fitted expression preserves the fundamental symmetries of the exact solution and captures the leading geometric dependence of the stability limit.

The resulting formulation is termed semi-analytical because it combines fully resolved (‘numerically exact’) solutions of the governing equations with an explicit semi-analytical representation that can be evaluated directly without further numerical solution. While the accuracy of the fit can, in principle, be increased by introducing higher-order terms, a low-order polynomial provides an excellent compromise between accuracy and simplicity, making the resulting expression particularly well suited for engineering applications such as spreadsheet-based process design.

The perturbation solution also predicts the peak temperature rise at the stability limit:

$$\Delta T_{\mathrm{crit}}=\vartheta_{\max,\mathrm{crit}}\;\varepsilon ,$$

where $\vartheta_{\max,\mathrm{crit}}=max_{x}\left(\vartheta_{\mathrm{crit}}\left(x\right)\right)$ is an 𝒪(1) shape factor that only depends on $\left(\sigma_{1},\sigma_{2},Bi_{d},Bi_{u}\right)$.

For a flat laminate with Dirichlet boundary conditions $\vartheta_{\max,\mathrm{crit}}=1.186$ [9]. However, for other curvatures, it is difficult to determine $\vartheta_{\max,\mathrm{crit}}$ accurately from the BVP because even a very small uncertainty in $\beta_{\mathrm{crit}}$ is strongly amplified in the peak-temperature estimate. This sensitivity makes the stepping method used to locate $\beta_{\mathrm{crit}}$ (see Appendix B) poorly suited for extracting $\Delta T_{\mathrm{crit}}$ directly from the perturbation equation.

Instead, the peak temperature at critical conditions is obtained from the full transient numerical model described in Appendix B, which exhibits much weaker sensitivity to small errors in the critical limit. The resulting values of $\Delta T_{\mathrm{crit}}$ are then fitted to the semi-analytical scaling law above, ensuring consistency between the perturbation theory and the ’exact’ numerical solution.

In summary, the perturbation method offers an intuitive description of thermal-runaway onset: geometry and surface heat transfer determine the shape of the stability boundary, while chemistry influences the critical Damköhler number through a simple exponential dependence. The resulting stability criterion in Equation (10) is concise, physically transparent, and highly accurate, as confirmed by comparison with fully coupled numerical simulations in the Section 3.

The discussion that follows examines the shape of the $\beta_{\mathrm{crit}}$ surface for two practically important heating configurations: (i) symmetric heating, where both surfaces have the same Biot number, and (ii) asymmetric heating, where one surface is strongly coupled to the surroundings and the other has a variable heat-transfer coefficient. These two cases cover the vast majority of industrial curing setups and illustrate the essential trends in how curvature and boundary conditions influence stability.

#### 2.2.1. Symmetric Heating

**Figure 2:**
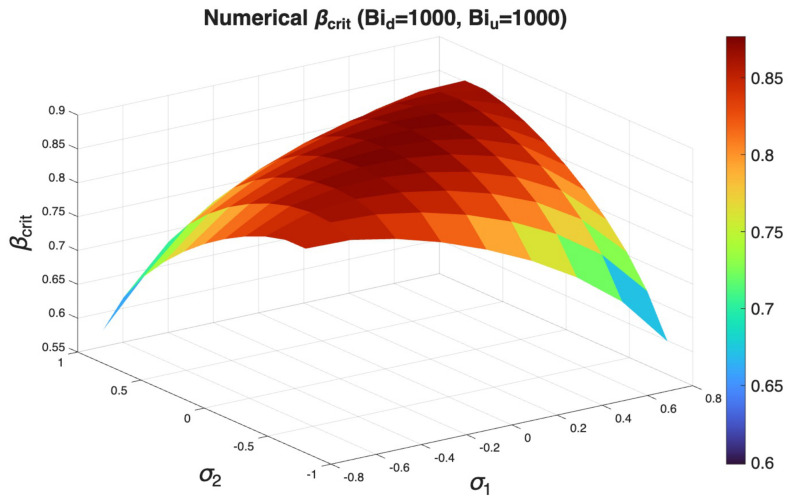
Critical stability limit $\beta_{\mathrm{crit}}$ as a function of the dimensionless principal curvatures $\sigma_{1}$ and $\sigma_{2}$ for symmetric heat-transfer conditions $\left(Bi_{u}=Bi_{d}=1000\right)$. The surface illustrates how the stability margin varies with curvature state, with anticlastic regions (opposite signs of $\sigma_{1}$ and $\sigma_{2}$) being more restrictive under symmetric heat-transfer conditions.

Figure 2 shows the stability surface $\beta_{\mathrm{crit}}\left(\sigma_{1},\sigma_{2}\right)$ for the case of symmetric heating, here illustrated with Bi=1000. Changing the Biot number shifts the overall height of the surface but does not alter its geometric shape. What is immediately apparent is the symmetry about the diagonal $\sigma_{1}=\sigma_{2}$. This reflects the exchange symmetry of the governing equations: interchanging the principal curvatures does not change the physical problem, and therefore $\beta_{\mathrm{crit}}\left(\sigma_{1},\sigma_{2}\right)=\beta_{\mathrm{crit}}\left(\sigma_{2},\sigma_{1}\right)$.

A second symmetry appears along the opposite diagonal, where $\left(\sigma_{1},\sigma_{2}\right)\to \left(-\sigma_{1},-\sigma_{2}\right)$. This transformation corresponds to reversing the choice of the positive thickness direction (x→−x), which changes the sign of the curvatures while leaving the physical geometry unchanged.

Under symmetric heating, the boundary conditions are identical on both surfaces, so this reversal does not affect the heat-transfer problem. Because the perturbation equation depends on the curvatures only through the combination $\left(\frac{\sigma_{1}}{1+\sigma_{1}x}+\frac{\sigma_{2}}{1+\sigma_{2}x}\right),$ the stability limit is therefore invariant under this transformation. Together, these two symmetries explain the characteristic appearance of the stability surface in Figure 2.

These symmetries strongly constrain the functional form of any semi-analytical expression for $\beta_{\mathrm{crit}}$. By rewriting the surface in terms of the curvature invariants

$$S=\sigma_{1}+\sigma_{2},\;P=\sigma_{1}\sigma_{2},$$

both symmetry conditions are satisfied automatically. Here S/2 is the dimensionless mean curvature and *P* is the dimensionless Gaussian-curvature invariant. This makes it possible to approximate the entire stability surface using a compact polynomial in *S* and *P*, which closely reproduces the numerical data:

(12) $$\beta_{\mathrm{crit}}\left(S,P,Bi\right)=c_{0}\left(Bi\right)\;+\;a\;S^{2}\;+\;b\;P,$$

**Figure 3:**
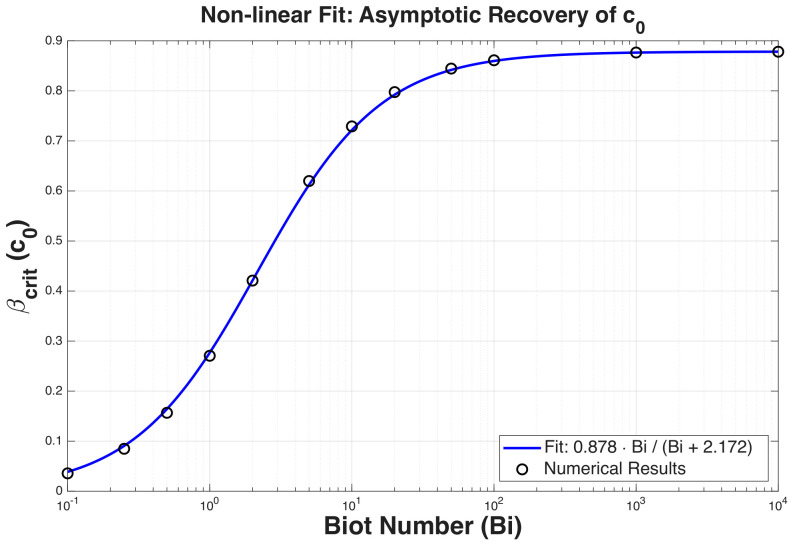
Dependence of the offset coefficient $c_{0}$ on the Biot number for symmetric boundary conditions. Symbols represent numerical results from the perturbation solution, while the solid line shows a fitted semi-analytical relation. The curve illustrates the rapid increase in stability with increasing heat-transfer efficiency and the saturation behaviour at high Biot numbers.

The coefficients a=0.121 and b=0.427 are obtained from a least-squares fit to the numerical data. While these parameters exhibit a weak Bi-dependency for low Biot numbers (Bi<5), they are treated here as constants to maintain the semi-analytical simplicity of the universal criterion. For typical laminate curvatures $\left(-0.4<\sigma_{1,2}<0.4\right)$, this approximation introduces an error of less than 1% for Bi≥5, and remains highly accurate even at Bi=1 with a maximum error of approximately 2.2%. Consequently, the curvature effects are decoupled from the surface heat transfer in the model, and only the offset term $c_{0}$ is required to vary with Bi:

(13) $$c_{0}\left(Bi\right)=\beta_{\mathrm{crit},\mathrm{Dirichlet}}\left(\frac{Bi}{Bi+K}\right),$$

where K=2.172 is a fitting parameter. The reference value $\beta_{\mathrm{crit},\mathrm{Dirichlet}}=0.87846$ represents the exact analytical stability limit for a flat slab $\left(\sigma_{1}=\sigma_{2}=0\right)$ under Dirichlet boundary conditions (Bi→∞) [9]. Equation (13) captures the dominant Bi-dependence with high fidelity, reflecting the rapid boundary-layer saturation observed as the process moves toward a diffusion-limited regime (see Figure 3).

Taken together, the symmetric-heating results provide a clear physical picture of how curvature modifies the onset of thermal runaway. The effect is not determined by curvature magnitude alone, but by the curvature state in the $\left(\sigma_{1},\sigma_{2}\right)$-plane. Under symmetric heating, anticlastic curvature states tend to reduce the stability margin, whereas synclastic states are less restrictive and may even increase the local stability factor within the fitted range. The Biot number, in contrast, primarily shifts the overall level of the stability surface by changing the efficiency of heat extraction at the boundaries.

The predicted peak temperature at the stability limit shows only a weak dependence on curvature but is strongly governed by the small parameter ε, and thus by the Arrhenius number and $T_{\infty }$.

From an engineering perspective, curvature primarily acts as a monotonic penalty on thermal stability, while the Biot number controls the overall heat-removal efficiency. The smoothness of the stability surface implies that moderate variations in curvature do not lead to abrupt changes in stability, improving predictability. In addition, the rapid saturation of $c_{0}\left(Bi\right)$ indicates diminishing returns from further increases in surface heat transfer, which is important for process design.

#### 2.2.2. Asymmetric Heating

For asymmetric heating conditions, where one surface is cooled more efficiently than the other $\left(Bi_{u}=1000,\;Bi_{d}=Bi<1000\right)$, the coordinate-flip symmetry of the system is broken. In the symmetric case, the stability limit was invariant under the simultaneous transformation of reversing the thickness coordinate (x→−x) and flipping the signs of the principal curvatures $\left(\left(\sigma_{1},\sigma_{2}\right)\to \left(-\sigma_{1},-\sigma_{2}\right)\right)$. In the asymmetric case, however, this transformation is no longer a symmetry of the problem because it swaps the positions of the well-cooled and poorly-cooled surfaces relative to the direction of curvature.

**Figure 4:**
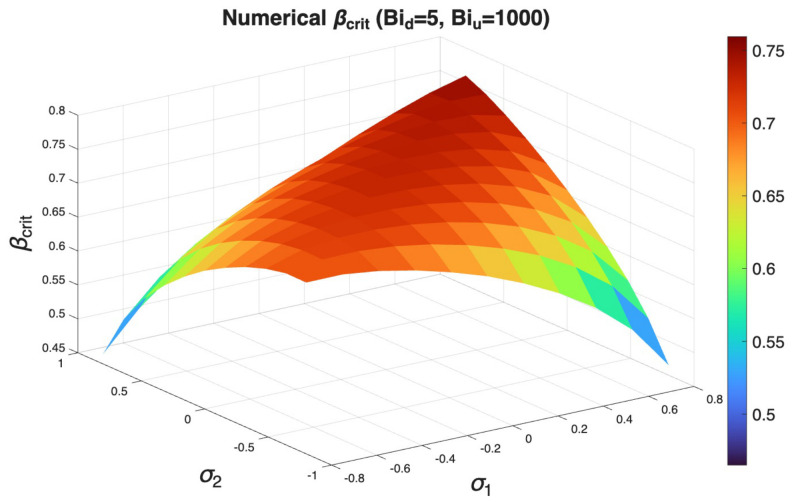
Critical stability limit $\beta_{\mathrm{crit}}$ as a function of $\sigma_{1}$ and $\sigma_{2}$ for asymmetric boundary conditions $\left(Bi_{u}=1000,\;Bi_{d}=5\right)$. The surface exhibits a clear tilt, showing that stability depends on the orientation of curvature relative to the weaker cooling surface. This highlights the influence of asymmetric heat-transfer conditions on the thermal runaway limit.

Consequently, the stability limit becomes sensitive to whether the laminate is curving “towards” or “away from” the side with inferior heat extraction. This manifests as a distinct “tilt” and distortion of the $\beta_{\mathrm{crit}}$ stability surface (see Figure 4), particularly at low Biot numbers (*Bi* < 10). In practical terms, this means that the same geometric shape may be more or less stable depending on which side faces the better-cooled boundary, highlighting the importance of part orientation during processing. In this regime, the onset of thermal runaway depends strongly on the orientation of the shell relative to the thermal bottleneck at the less-cooled surface. As the cooling efficiency increases toward *Bi* ≈10, this sensitivity to orientation diminishes, and the stability surface eventually recovers the symmetric characteristics of the Bi→∞ limit.

To capture this physical distortion mathematically, the semi-analytical fitting polynomial must be modified. Specifically, an additional linear term in the curvature sum *S* is required to account for the first-order sensitivity to curvature direction that appears when the cooling is unbalanced:

(14) $$\beta_{\mathrm{crit}}\left(S,P,Bi\right)=c_{0}\left(Bi\right)+L\left(Bi\right)\;S+a\left(Bi\right)\;S^{2}+b\left(Bi\right)\;P$$

**Figure 5:**
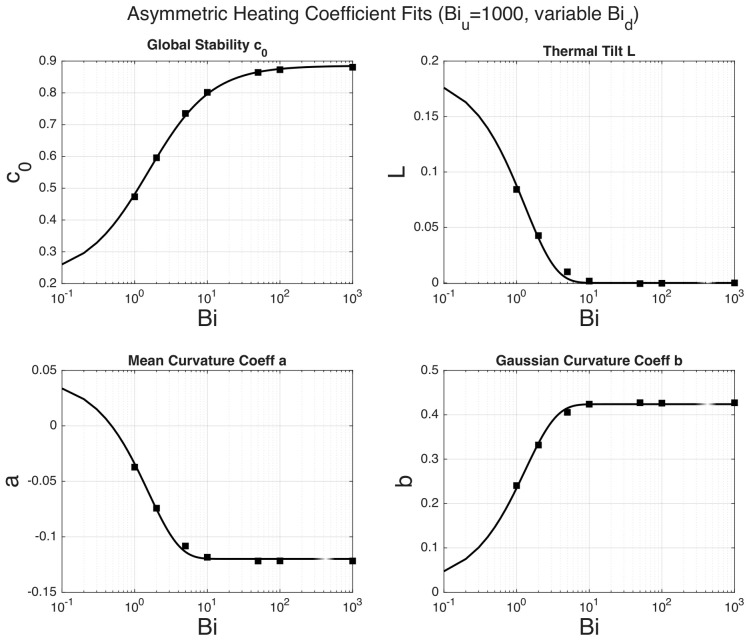
Fitted coefficients of the polynomial representation of $\beta_{\mathrm{crit}}$ for asymmetric boundary conditions as a function of Biot number. The smooth variation of the coefficients shows how the effect of asymmetry diminishes as heat-transfer conditions become more balanced.

In this asymmetric regime, all four coefficients $\left(c_{0},L,a,b\right)$ exhibit a dependency on the Biot number. The linear coefficient L(Bi) explicitly quantifies the “tilt” of the surface, while the remaining terms describe the evolution of the surface height and curvature sensitivity. These dependencies are accurately captured by the following fitting functions:

(15a) $$c_{0}\left(Bi\right)=\frac{c_{1}\;Bi+c_{2}\;c_{3}}{Bi+c_{3}},$$

(15b) $$L\left(Bi\right)=L_{1}\exp\;\left(-\frac{Bi}{L_{2}}\right),$$

(15c) $$a\left(Bi\right)=a_{1}+a_{2}\exp\;\left(-\frac{Bi}{a_{3}}\right),$$

(15d) $$b\left(Bi\right)=b_{1}-b_{2}\exp\;\left(-\frac{Bi}{b_{3}}\right).$$

with the optimised coefficients listed in Table 1. These functions reproduce the $\beta_{\mathrm{crit}}$ surface for all fitted data with errors well below one percent (see Figure 5).

The asymmetric-heating results reveal several important differences compared to the symmetric case. The most prominent is that unequal boundary conditions introduce a directional bias into the heat-removal capability of the shell. This appears as a characteristic “tilt” of the stability surface, captured by the coefficient L(Bi), which shifts the stability margin depending on whether the dominant curvature faces the hotter or the better-cooled surface. For engineers, this has a clear implication: orientation of the heating matters. A curved part with one side poorly cooled is inherently more vulnerable to runaway when its convex face is exposed to the weaker boundary condition.

Despite this asymmetry, many trends remain consistent. The dimensionless mean-curvature invariant S/2 and the dimensionless Gaussian-curvature invariant *P* still control the overall curvature dependence through the coefficients a(Bi) and b(Bi), and these vary smoothly with Bi. Once the poorly cooled face reaches moderate heat-transfer efficiency, the influence of asymmetry decreases rapidly, mirroring the saturation behaviour discussed above. This means that in many practical setups, especially autoclave or oven curing where one surface is strongly coupled to the environment, only modest improvements to the weaker surface are needed to bring the system close to the more stable symmetric-heating case.

Despite the asymmetry, the stability surface remains smooth and predictable across the parameter space. While unequal boundary conditions reduce the overall stability margin, the effect is systematic, allowing engineers to quantify the influence of part orientation, tooling contact, and insulation without resorting to full numerical simulations.

#### 2.2.3. General Boundary Conditions

While the symmetric and strongly asymmetric configurations discussed above capture the essential physical phenomena, a general heating scenario may involve two arbitrary, unequal Biot numbers. Conceptually, the stability surface for such a general case can be understood as combining the two behaviours identified above. If one initially assumes a symmetric state based on the higher of the two Biot numbers, the overall elevation of the $\beta_{\mathrm{crit}}$ surface is correspondingly reduced. Subsequently reducing the remaining surface’s Biot number to its true, lower value breaks the symmetry, introducing the characteristic “tilt” and, at sufficiently low values, the flattening observed in the asymmetric case.

Rather than developing an exhaustive semi-analytical formulation for all possible $\left(Bi_{u},Bi_{d}\right)$ combinations, a computational approach is recommended for general conditions. The software provided in the Supplementary Material (detailed in Appendix B) allows the user to input any arbitrary pair of Biot numbers to automatically generate a custom polynomial fit for $\beta_{\mathrm{crit}}$. Once this specific fit is generated, the thermal stability of the part can be straightforwardly evaluated using the theoretical framework $\left(Da_{\mathrm{crit}},\Delta T_{\max},\;\mathrm{and}\;w_{\max}\right)$ established in the preceding sections.

### 2.3. Numerical Methods (Summary)

The perturbation boundary-value problem for $\beta_{\mathrm{crit}}$ is solved using MATLAB’s bvp4c solver combined with a continuation strategy in β (details in Appendix B). For each parameter set, a sequence of stationary solutions is computed for increasing values of β until the solver can no longer satisfy the prescribed error tolerances within the allowed mesh resolution. The corresponding value is identified as $\beta_{\mathrm{crit}}$, i.e., the limit of existence of a converged stationary solution.

The bvp4c solver employs an adaptive collocation scheme with automatic mesh refinement to meet the specified error tolerances. In the present work, a sufficiently large maximum mesh size (typically up to $10^{4}$ grid points) is used to ensure that the solution is fully resolved even near the critical limit. The solutions remain smooth over the entire domain, and no convergence issues are observed within the stability region.

The fully coupled transient 1D thermo-kinetic model used for validation of the perturbation model is discretised by a second-order cell-centred finite-volume method (method of lines) and integrated with MATLAB’s ode15s using a relative tolerance of $10^{-8}$ (Appendix B). A grid-convergence study with Richardson extrapolation indicates that the production grid yields relative errors below $2\times 10^{-4}$ for the peak temperature, which is negligible compared to the model fitting error.

Thermal runaway in the numerical solution is classified objectively using the sign of the peak second time derivative of a virtual sensor temperature at the hottest node, and $Da_{\mathrm{crit}}$ is located using MATLAB’s fzero solver (Appendix B).

The computational effort required to generate the numerical results is modest. The $\beta_{\mathrm{crit}}$ surface is obtained from a sequence of boundary-value problem solutions and can be computed within a few minutes on a standard laptop. Individual transient simulations of the fully coupled thermo-kinetic model are fast, with typical run times of a few seconds for a given parameter set. The evaluation of the critical Damköhler number $Da_{\mathrm{crit}}$ requires multiple transient simulations, but the total computational time remains below one minute.

The complete MATLAB (R2025a) source code used to generate the results is provided as Supplementary Material to ensure reproducibility.

## 3. Results

This section validates the perturbation-based predictions against high-precision numerical solutions of the fully coupled thermal-kinetic problem (Equations (4) and (5)). The standalone transient solver (Appendix B) resolves the through-thickness energy balance with Robin boundary conditions and autocatalytic kinetics without the simplifying assumptions used in the perturbation analysis. A grid-convergence study with Richardson extrapolation showed that, for N=100 control volumes, the relative error was below $2\times 10^{-4}$.

An objective classification scheme (Appendix B) was used to distinguish sub-critical from super-critical solutions based on the temperature-time history at an internal sensor point. Coupled with a bracketed search in the Damköhler number, this made it possible to determine the critical Damköhler number $Da_{\mathrm{crit}}$ for any prescribed set of parameters. These “numerically exact” solution results are compared with the semi-analytical perturbation predictions, focusing on four aspects: (i) the transient behaviour near criticality, (ii) the geometric scaling through $\beta_{\mathrm{crit}}$, (iii) the Arrhenius/temperature scaling in the prefactor and the predicted peak temperature, and (iv) the validity of the locally one-dimensional approximation with respect to lateral heat transport.

The remainder of the section is organised as follows: (i) the characteristics of the transient temperature response near critical conditions; (ii) geometrical validation of the $\beta_{\mathrm{crit}}\left(S,P\right)$ surface and its polynomial representation; (iii) chemical/temperature calibration and generalisation across kinetic exponents; and (iv) assessment of lateral heat transport using the analytical and quasi-three-dimensional flux-ratio analyses.

### 3.1. Characteristics of the Temperature History at Critical Conditions

When the Damköhler number exceeds its critical value, heat generation can no longer be balanced by heat removal within the reduced stability model, and the temperature response becomes self-accelerating. However, the qualitative nature of the resulting temperature rise is dependent on the Arrhenius number $A_{r}$, which dictates whether the runaway is “mild” or “explosive.”

**Figure 6:**
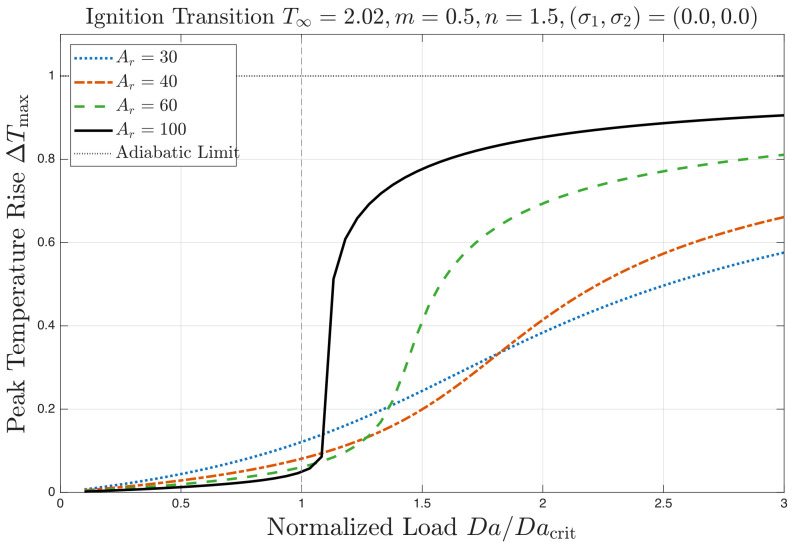
S-curve behaviour of the peak temperature rise in a flat laminate ($\sigma_{1}=\sigma_{2}=0$) for different Arrhenius numbers. The Damköhler number is normalised by its critical value, with the vertical dashed line marking the stability limit. For low Arrhenius numbers the temperature increases gradually, while higher values lead to a more pronounced S-shape, illustrating the change from mild to ignition-like thermal runaway behaviour.

**Figure 7:**
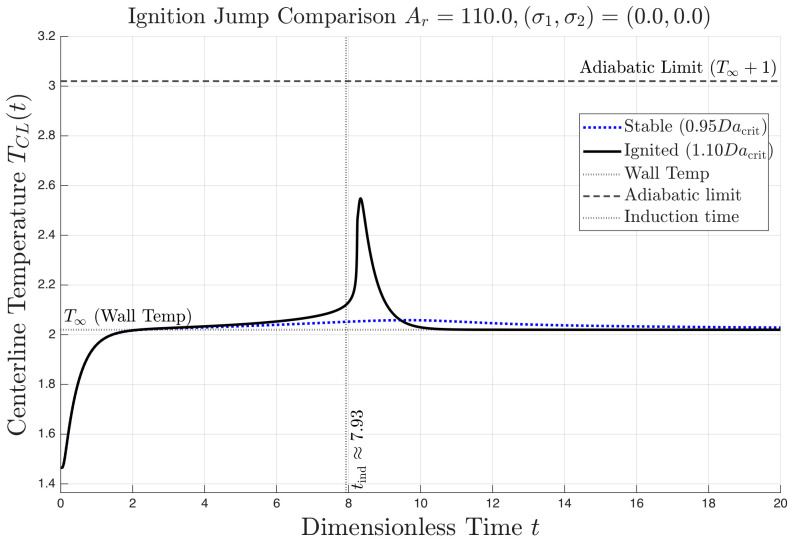
Temperature evolution at high Arrhenius number, comparing sub-critical and super-critical conditions. The super-critical case exhibits a long induction period followed by rapid temperature rise, characteristic of ignition-like runaway, while the sub-critical case remains stable. This illustrates the strong sensitivity of the system to small changes in the Damköhler number near the stability limit.

- Mild (gradual) runaway—low $A_{r}$: For Arrhenius numbers below a practical threshold, defined here by
  (16) $$A_{r,\mathrm{crit}}\;\approx \;16.67\;T_{\infty }\;\left(1+T_{\infty }\right),$$
  the peak temperature increases gradually with Da, exhibiting a nearly linear dependence on Da up to and beyond the stability limit (see $A_{r}=30$ in Figure 6). In this regime, crossing $Da_{\mathrm{crit}}$ does not trigger a catastrophic jump, but rather a gradual increase in the exotherm.
- Ignition-like (explosive) runaway—high $A_{r}$: For Arrhenius numbers exceeding the threshold in Equation (16), the system behaviour changes character. Sub-critical cases remain stable, but once $Da_{\mathrm{crit}}$ is reached the system exhibits a long induction period followed by a rapid temperature rise toward the adiabatic limit (see $A_{r}=100$ in Figure 7), a behaviour closely resembling ignition in combustible mixtures [14].

At the critical limit ($Da/Da_{\mathrm{crit}}=1$), the numerical peak temperature rise $\Delta T_{\max}$ follows the scaling $\Delta T_{\max}\propto T_{\infty }^{2}/A_{r}$ predicted by the perturbation analysis. For the cases illustrated in Figure 6 ($A_{r}=30,40,60,100$), the numerical peak values at critical condition are approximately 0.12,0.08,0.06 and 0.05, respectively. These values are slightly lower than the absolute peaks predicted by the perturbation analysis, but the ratios of the magnitudes are consistent with the theory.

The higher peak temperatures in the perturbation model compared to the numerical simulations stem from the simplified kinetics used in the perturbation model, specifically the replacement of the kinetic function f(α) by a constant, which overestimates the reaction rate throughout the cure. By neglecting reactant consumption and the specific (m,n)-order kinetics, perturbation theory provides a conservative upper bound for the temperature rise. In contrast, the numerical model captures the reduction in reaction rate as conversion increases, leading to the slightly lower peak temperatures observed here.

The threshold in Equation (16) represents a Semenov-type criterion [14] adapted to the current shell geometry and boundary conditions. Its approximate numerical value was determined by identifying the lowest $A_{r}$ at which the $\Delta T_{\max}$ vs. Da response transitions from a continuous curve to a nearly discontinuous jump when exceeding $Da_{\mathrm{crit}}$. Although classical Semenov theory is derived for lumped systems, this empirically calibrated form successfully captures the observed bifurcation in behaviour, explaining why minor process variations in Da can be benign at low $A_{r}$ but catastrophic at high $A_{r}$.

### 3.2. Validation of the Curvature-Chemistry Separation

The validation of the separation of curvature effects from the chemistry $Da_{\mathrm{crit}}\propto \beta_{\mathrm{crit}}\left(\sigma_{1},\sigma_{2},Bi_{d},Bi_{u}\right)$ was done with a fixed chemistry, $(A_{r}=32.4,\;T_{\infty }=2.335,$ m=0.313, n=1.66), taken from a reported 120 °C epoxy system [4]. Anchoring the study to a documented material avoids unphysical parameter combinations. This choice does not bias the geometric results because $\beta_{\mathrm{crit}}$ is independent of chemistry; chemical effects enter only when converting to $Da_{\mathrm{crit}}$.

In what follows, the geometry-Biot dependence of the stability limit $\beta_{\mathrm{crit}}$ is taken directly from the polynomial fits in Equations (12) and (14) from Section 2. The resulting global stability limit $Da_{\mathrm{crit}}$ defined by Equation (10) was fitted to the numerical results for the different heating options using the kinetic scaling factor $C_{\alpha }$ as the only fitting parameter.

**Figure 8:**
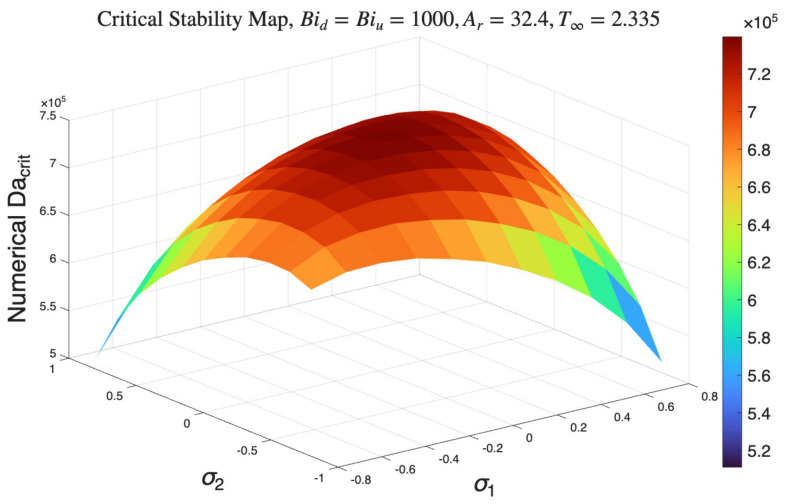
Numerically computed critical Damköhler number $Da_{\mathrm{crit}}$ as a function of the principal curvatures for symmetric high-Biot-number conditions. The surface exhibits the same symmetry and geometric trends predicted by the perturbation model, confirming that the stability margin is governed by the curvature state in the $\left(\sigma_{1},\sigma_{2}\right)$-plane. The close agreement demonstrates that the semi-analytical framework accurately captures the geometry dependence of the stability limit.

We first consider symmetric heating at a high Biot number ($Bi_{d}=Bi_{u}=1000$). Figure 8 shows the numerically computed stability surface $Da_{\mathrm{crit}}\left(\sigma_{1},\sigma_{2}\right)$. The surface exhibits the same symmetries as the $\beta_{\mathrm{crit}}\left(\sigma_{1},\sigma_{2}\right)$ surface from the perturbation analysis, namely invariance under exchange $\left(\sigma_{1},\sigma_{2}\right)\mapsto \left(\sigma_{2},\sigma_{1}\right)$ and, under symmetric boundary conditions, invariance under the combined transformation $\left(\sigma_{1},\sigma_{2},x\right)\mapsto \left(-\sigma_{1},-\sigma_{2},-x\right)$. The one-parameter fit yields $C_{\alpha }=0.213$, about 50% of maxf(α) for the chosen kinetics, indicating that the conservative choice $C_{\alpha }=\max\left(f\left(\alpha \right)\right)$ in the Theory section is unnecessarily strict. The RMS relative error for the fit is 1.8% with a maximum deviation of 4.6% over the whole $\left(\sigma_{1},\sigma_{2}\right)$-plane.

For the moderate-convection case $Bi_{d}=Bi_{u}=5$, the same one-parameter fitting procedure yields an almost identical coefficient, $C_{\alpha }=0.208$, with an RMS relative error of 2.4% and a maximum deviation of 6.4%. The closeness of $C_{\alpha }$ to the high-Bi fit (0.213) confirms that the chemistry/process scaling is effectively independent of surface heat-transfer strength, while the Biot number acts primarily through the geometry–boundary factor $\beta_{\mathrm{crit}}$ (i.e., a uniform vertical shift of the surface without altering its shape). In other words, even away from the near-Dirichlet limit, the predicted separation of geometry+boundaries from chemistry+temperature holds: the semi-analytical $\beta_{\mathrm{crit}}\left(S,P\right)$ surface from the perturbation solution can be used *as is*, and a single $C_{\alpha }$ brings the model into close agreement with the full numerical results.

For asymmetric heating, the outer surface is taken nearly isothermal $\left(Bi_{u}=1000\right)$ while the inner surface has finite thermal resistance $\left(Bi_{d}\geq 0\right)$. The computed stability surfaces again exhibit the symmetries predicted by the perturbation analysis, including the expected tilt along the mean-curvature direction $S=\sigma_{1}+\sigma_{2}$ for small $Bi_{d}$. The semi-analytical surface $\beta_{\mathrm{crit}}\left(S,P;Bi_{d},Bi_{u}\right)$ from the perturbation analysis is kept unchanged and only the single kinetic factor $C_{\alpha }$ is fitted for each $Bi_{d}$.

Across the full range of asymmetric boundary conditions, the fitted values of $C_{\alpha }$ remain tightly clustered within the interval $0.21\leq C_{\alpha }\leq 0.22$ for $\left(Bi_{u}=1000,Bi_{d}\leq 100\right)$, indicating strong robustness of the chemistry–temperature scaling. For practically relevant heat-transfer conditions, the model shows very good quantitative agreement with the full numerical solution. For example, at $Bi_{d}=1$, the RMS relative error is only 3.3%, decreasing further to below 2% for $Bi_{d}\geq 10$ (Table 2).

The largest deviations occur in the limiting case of a perfectly insulated inner surface $\left(Bi_{d}=0\right)$, where the RMS relative error is 11.6% and the maximum deviation reaches 55.9%. However, this case represents an extreme boundary condition with very weak heat extraction, and the large relative error must be interpreted with care. For $Bi_{d}=0$, the range of $Da_{\mathrm{crit}}$ values spans more than an order of magnitude, such that relatively uniform absolute deviations translate into larger percentage errors at the lower end of the range.

**Figure 9:**
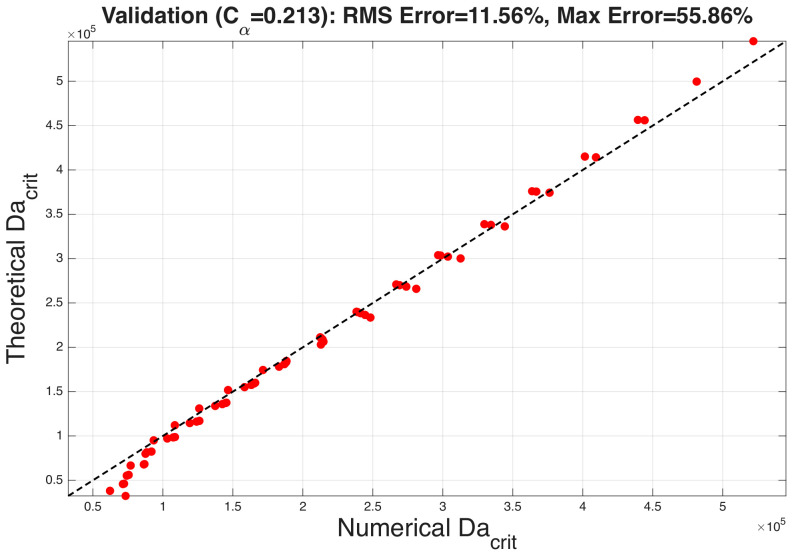
Parity plot comparing the critical Damköhler number obtained from the fully coupled model and the perturbation-based prediction for asymmetric boundary conditions. The close clustering around the y=x line demonstrates good agreement across the full range, indicating that the model accurately captures the stability trends even under strongly asymmetric cooling.

Consistent with this interpretation, the parity plot in Figure 9 shows a tight clustering around the y=x line across the full dataset, indicating that the model accurately captures the underlying trend and scaling behaviour even in this extreme case. The largest deviations are observed for the most extreme curvature values included in the analysis, which lie at the upper bound of the considered parameter range.

Overall, the results confirm that the geometry–boundary contribution can be taken directly from the perturbation BVP, while the chemistry–temperature influence is captured by a single scalar $C_{\alpha }$ (here ≈0.215) without re-tuning the geometric fit.

Across all symmetric and asymmetric cases, the best-fit $C_{\alpha }$ remains nearly constant (≈0.21–0.22) over the entire $\left(\sigma_{1},\sigma_{2}\right)$ grid and Biot-number range, reinforcing that geometry/boundary effects are captured by $\beta_{\mathrm{crit}}$ while chemistry is well represented by a single scalar $C_{\alpha }$.

### 3.3. Chemical/Temperature Calibration

Having confirmed that the surface-curvature and boundary heat-transfer dependence of the stability limit is accurately captured by the perturbation-theory parameter $\beta_{\mathrm{crit}}\left(S,P,Bi\right)$, we now turn to the calibration of the chemical prefactor $C_{\alpha }$. This factor represents the effective exothermic strength of the reaction and appears multiplicatively in the semi-analytical reduced-order criterion for the critical Damköhler number. The objective of this section is to determine whether a single constant value of $C_{\alpha }$ is sufficient to represent the chemical contribution across a broad range of Arrhenius numbers and surface temperatures.

#### Calibration over $A_{r}$ and $T_{\infty }$

To isolate chemical effects from geometry and boundary conditions, all calibration simulations were performed for a fixed configuration: vanishing curvature $\left(\sigma_{1}=\sigma_{2}=0\right)$, symmetric heating with Bi=1000, and fixed kinetic exponents (m=0.313,n=1.66), identical to those used in the geometrical validation. The validated separation between geometry/boundary heat-transfer effects and chemistry/temperature effects ensures that this restriction does not bias the calibration.

**Figure 10:**
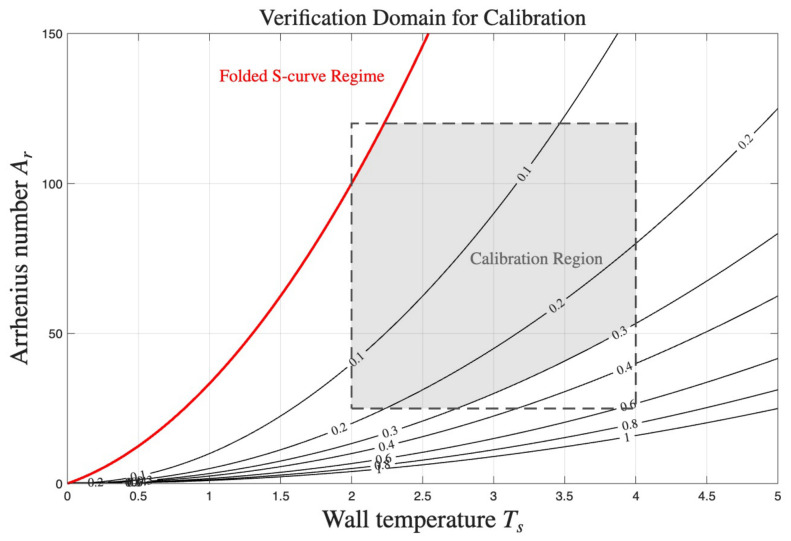
Calibration domain in the $\left(A_{r},T_{\infty }\right)$ plane. The shaded region defines the parameter range used for model calibration, bounded by limits on the perturbation validity and by exclusion of highly unstable conditions. The isolines indicate the scaling parameter ε, which governs the magnitude of the temperature perturbation.

The calibration spanned a rectangular domain in the $\left(A_{r},\;T_{\infty }\right)$ plane, $25\leq A_{r}\leq 120,$ $\;2\leq T_{\infty }\leq 4$, subject to two constraints: (i) ε≤0.3, ensuring the perturbation solution remains accurate, and (ii) exclusion of the region $A_{r}>16.67\;T_{\infty }\left(1+T_{\infty }\right)$, where the system is prone to a sudden, catastrophic jump in temperature and severe overheating for even small excursions above $Da_{\mathrm{crit}}$. The resulting admissible domain is shown in Figure 10.

**Figure 11:**
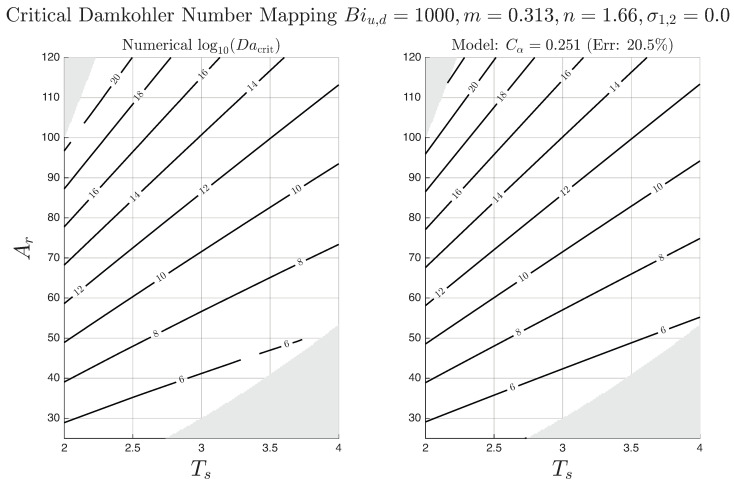
Comparison between the numerically computed critical Damköhler number and the perturbation-based prediction over the $\left(A_{r},T_{\infty }\right)$ domain. The close alignment of isolines demonstrates that the model captures the correct scaling with Arrhenius number and processing temperature, despite moderate quantitative deviations.

Within the calibration domain, the critical Damköhler number $Da_{\mathrm{crit}}$ was extracted from fully coupled transient simulations using the objective classification scheme of Appendix B. These numerical “truth” values were then compared with the perturbation- theory prediction. A least-squares fit over the entire $\left(A_{r},T_{\infty }\right)$ region gives $C_{\alpha }=0.251$, slightly larger than the value obtained in the geometrical study (≈0.213). The fitted constant represents approximately 59% of max(f(α)) for these kinetics and yields an average relative error of 20.5% in $Da_{\mathrm{crit}}$. While this level of deviation may appear significant in absolute terms, it should be noted that the perturbation-based model correctly reproduces the shape and scaling of the stability boundary across the entire parameter space. In practice, this level of accuracy is comparable to or smaller than typical uncertainties in kinetic parameters and heat-transfer coefficients, and the model therefore remains well suited for design screening and process optimisation. Figure 11 shows that, despite the moderate absolute error, the shape of the isolines and the combined Arrhenius–temperature scaling are reproduced extremely well.

**Figure 12:**
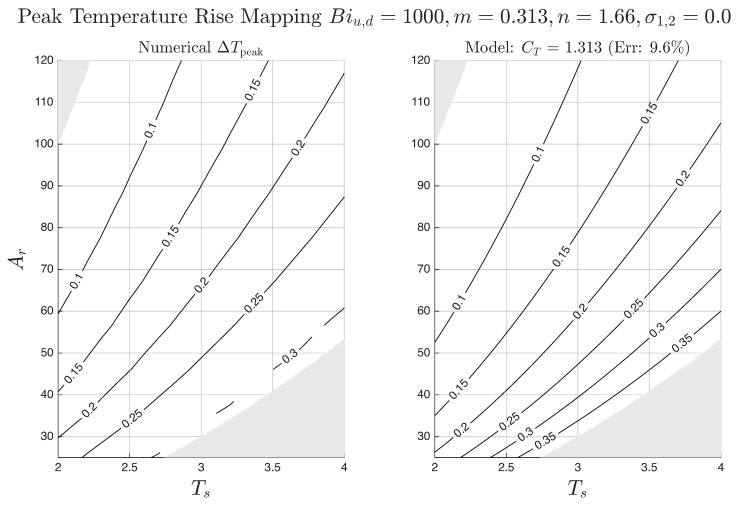
Comparison of the peak temperature rise at critical conditions between the numerical solution and the perturbation model. The isolines follow the scaling predicted by the perturbation analysis, showing that the temperature rise is primarily governed by the parameter ε.

A similar fitting procedure was applied to the predicted peak temperature at criticality, yielding an optimal temperature-scaling factor $C_{T}=1.313$, with an average relative error of approximately 9.5%. As illustrated in Figure 12, the critical-temperature isolines align closely with those of constant ε in Figure 10, consistent with the theoretical structure of the perturbation solution.

Overall, the comparison indicates that the semi-analytical perturbation framework captures the dominant chemical and thermal scaling across a broad parameter space. The fitted constants $C_{\alpha }$ and $C_{T}$ are effectively uniform over the calibration domain, and the remaining discrepancies are of the magnitude anticipated for a first-order perturbation approximation. Most importantly, the isoline alignment confirms that the perturbation solution retains the correct topology of the stability boundary, validating its use as a reduced-order predictive model for thermal runaway.

### 3.4. Generalisation Across Kinetics

Autocatalytic reaction models of the form $f\left(\alpha \right)=\alpha^{m}{\left(1-\alpha \right)}^{n}$ are widely used to describe thermoset curing [12]. However, only a restricted subset of exponent pairs (m,n) produces conversion-rate profiles that resemble experimentally observed behaviour. In particular, very small values of *m* or unusually large values of *n* cause the reaction rate to become highly skewed: the peak of f(α) shifts unrealistically close to either α=0 or α=1, the maximum value $f_{\max}$ becomes excessively sharp, and the overall curve loses the broad, single-hump shape characteristic of epoxy and polyester systems. Such behaviours are inconsistent with DSC measurements reported in the literature and lead to runaway thresholds that do not represent industrial thermosets.

To ensure physically meaningful kinetics while still covering a broad range of autocatalytic behaviours, we therefore restrict attention to

0.3≤m≤0.5,1.0≤n≤1.7,

a domain consistent with reported kinetic fits for common resin families and free from pathological rate-curve shapes. Within this region, f(α) remains single-peaked, moderately skewed, and shows the characteristic early-stage acceleration followed by depletion-driven decay. Most commercial resin systems fall well within this window, and the associated rate curves differ primarily in width and skewness rather than in qualitative shape. Should values outside this region be required, a new calibration can easily be performed using the software provided in the Supplementary Material.

#### 3.4.1. Dense Sweep and Global Response-Surface Fit

A full systematic sweep across the (m,n) domain was carried out to obtain a smooth functional form for $C_{\alpha }$. Each (m,n) pair was calibrated against a dense grid in $\left(A_{r},T_{\infty }\right)$, yielding a corresponding value of $C_{\alpha }$ that was used to define a damping factor that relates $C_{\alpha }$ to the maximum of the kinetic function:

$$K\left(m,n\right)=\frac{C_{\alpha }}{f_{\max}\left(m,n\right)},$$

where $f_{\max}=\max_{\alpha }f\left(\alpha \right)$. The sweep used a 5×5 grid in (m,n) and, for each point, a 21×10 grid in $\left(T_{\infty },A_{r}\right)$, with the fitting procedure for $C_{\alpha }$ identical to that used for the chemical calibration.

The resulting optimised parameters $C_{\alpha }$ and $C_{T}$, their associated relative errors for $Da_{\mathrm{crit}}$ and $\Delta T_{\max}$, and the normalised damping factor

$$K=\frac{C_{\alpha }}{f_{\max}}$$

are summarised in Table 3. The consistency of $C_{\alpha }$ and $C_{T}$ across these widely separated kinetic exponents confirms that the calibration procedure is consistent across the parameter range.

The resulting K(m,n) data were fitted to a low-dimensional polynomial response surface:

(17) $$K\left(m,n\right)=p_{00}+p_{10}m+p_{01}n+p_{20}m^{2}+p_{11}mn,$$

with coefficients

$$\begin{array}{ccc} p_{00}=0.8266, & p_{10}=-0.6373, & p_{01}=-0.1630,\\ p_{20}=2.5335, & p_{11}=-0.0204, & \end{array}$$

yielding $R^{2}=0.9993$.

A similar fit was obtained for the temperature-scaling factor:

(18) $$C_{T}\left(m,n\right)=p_{00}+p_{10}m+p_{01}n+p_{20}m^{2}+p_{11}mn+p_{02}n^{2},$$

with

$$\begin{array}{ccc} p_{00}=1.8510, & p_{10}=-1.2733, & p_{01}=-0.0315,\\ p_{20}=-2.9990, & p_{11}=0.9710, & p_{02}=-0.1082, \end{array}$$

and $R^{2}=1.0000$.

#### 3.4.2. Final Predictive Forms

For operational use, the effective rate constant is taken as

(19) $$C_{\alpha }=K\left(m,n\right)\;f\left(\alpha_{\max}\right),$$

with

(20) $$\alpha_{\max}=\frac{m}{m+n},\;f\left(\alpha_{\max}\right)={\left(\frac{m}{m+n}\right)}^{m}{\left(\frac{n}{m+n}\right)}^{n}.$$

Within the calibration region

$$0.3\leq m\leq 0.5,\;1.0\leq n\leq 1.7,\;25\leq A_{r}\leq 120,\;2\leq T_{\infty }\leq 4,\;\sigma_{1,2}\in \left[-0.8,0.8\right],$$

subject to the constraint $\varepsilon =T_{\infty }^{2}/A_{r}\leq 0.3$ and excluding the folded S-curve regime, the damping factor remains bounded between 0.57 and 0.97. Outside this region, *K* should be taken as capped at K=1.0, corresponding to the limiting case $C_{\alpha }=f_{\max}$, which is guaranteed to yield a critical limit on the safe side of the value obtained from the exact solution.

Substituting (19) into the semi-analytical stability criterion (Equation (10)) yields:

(21) $$Da_{\mathrm{crit}}\left(\sigma_{1},\sigma_{2},Bi_{d},Bi_{u},m,n,T_{\infty },A_{r}\right)=\frac{\beta_{\mathrm{crit}}\left(\sigma_{1},\sigma_{2},Bi_{d},Bi_{u}\right)}{K\left(m,n\right)\;f\left(\alpha_{\max}\right)}\;\frac{T_{\infty }^{2}}{A_{r}}\;\exp\;\left(\frac{A_{r}}{T_{\infty }}\right),$$

where K(m,n) and $f(\alpha_{\max})$ are defined in Equations (17) and (20), and $\beta_{\mathrm{crit}}\left(\sigma_{1},\sigma_{2},Bi_{d},Bi_{u}\right)$ is defined in Equations (12) and (14) for symmetric and asymmetric heating respectively.

#### 3.4.3. Temperature-Rise Generalisation

An analogous generalisation applies to the maximum temperature rise $\Delta T_{\max}$ at $Da=Da_{\mathrm{crit}}$. As shown in Table 3, the factor $C_{T}$ varies systematically with (m,n), ranging from approximately 0.81 to 1.36. These variations are consistent with perturbation-theory expectations: changes in the skewness of f(α) alter the heat-release rate, but $C_{T}$ remains 𝒪(1) across all practical cases. The relative error in $\Delta T_{\max}$ remained between 4.6% and 14% across all tested exponent pairs.

Fitting the numerical data to $C_{T}\left(m,n\right)$ provides a smooth functional dependence that reproduces numerical calibrations with high fidelity. Although the model could theoretically be refined by incorporating explicit semi-analytical corrections for local curvatures $\left(\sigma_{1},\sigma_{2}\right)$ and Biot numbers $\left(Bi_{d},Bi_{u}\right)$, the current formulation already exhibits excellent accuracy. Therefore, this simpler approach was retained to prioritize ease of practical implementation without compromising predictive reliability. The resulting expression for the peak temperature rise is

(22) $$\Delta T_{\max}\left(\sigma_{1},\sigma_{2},Bi_{d},Bi_{u},m,n,T_{\infty },A_{r}\right)=C_{T}\left(m,n\right)\;\frac{T_{\infty }^{2}}{A_{r}},$$

using the fit for $C_{T}\left(m,n\right)$ from Equation (18).

### 3.5. Verification of Global Accuracy

To further assess the global accuracy of the fully assembled model, a final validation series was conducted using a fractional factorial experimental design [15]. The parameter space was bounded by the limits of extreme industrial part geometries $\left(\sigma_{1,2}=\pm 0.4\right)$, spanning flat, strongly synclastic, and strongly anticlastic shells. Boundary conditions were restricted to either fully symmetric cooling $\left(Bi_{d}=Bi_{u}=1000\right)$ or heavily asymmetric cooling $\left(Bi_{d}=10,Bi_{u}=1000\right)$. The kinetic exponents were tested at the boundaries of the admissible domain (m∈[0.3,0.5],n∈[1.0,1.7]).

An 8-run orthogonal test matrix was constructed to sample the corners of this multidimensional space without redundant geometric configurations. For each of the 8 cases, a direct numerical calibration sweep over the $\left(A_{r},T_{\infty }\right)$ plane was performed to extract the true values of $C_{\alpha }$ and $C_{T}$. These were then compared against predictions of the semi-analytical global model. Because $Da_{\mathrm{crit}}\propto 1/C_{\alpha }$ and $\Delta T_{\max}\propto C_{T}$, the relative errors in these prefactors directly represent the model’s predictive error for the runaway threshold and exotherm severity. The results, summarized in Table 4, show that the global equations successfully capture the compounding effects of asymmetric curvature, heat transfer, and complex kinetics.

### 3.6. Assessment of Lateral Heat Transport and Validity of the Locally One-Dimensional Approximation

The theoretical model developed in this work assumes that heat transport is locally dominated by through-thickness conduction, such that lateral conduction along the laminate surface can be neglected. This assumption is central to the reduction of the shell problem to a locally one-dimensional formulation. Appendix C assesses its validity by deriving an analytical flux-ratio criterion and by comparing it with quasi-three-dimensional numerical flux evaluations for representative shell geometries.

The key point is that the relevant lateral quantity is not the absolute heat crossing an individual lateral face, but the net warming or cooling effect produced by lateral conduction within a local control volume. In the curvilinear control-volume balance, the local footprint area cancels, and the omitted lateral contribution reduces to a surface divergence governed by the Laplace–Beltrami operator. The resulting diagnostic is the conductive flux ratio

$$R_{\mathrm{flux}}=\left|\frac{{\bar{Q}}_{\mathrm{lat},\mathrm{net}}}{{\bar{Q}}_{\mathrm{trans}}}\right|,$$

where ${\bar{Q}}_{\mathrm{lat},\mathrm{net}}$ is the net lateral conductive divergence and ${\bar{Q}}_{\mathrm{trans}}$ is the corresponding transverse conductive contribution. Values of $R_{\mathrm{flux}}\ll 1$ indicate that lateral conduction has only a minor influence on the local heat balance.

A particularly noteworthy outcome of the analysis is that the analytical flux-ratio estimate is independent of the magnitude of the chemical heat source. Apart from the conductivity anisotropy factor and an order-unity through-thickness profile factor, it is controlled by the shell geometry and by the local value of the critical stability parameter $\beta_{\mathrm{crit}}$, which itself is determined by geometry and boundary conditions through the local Biot number. The analytical estimate separates naturally into a through-thickness profile factor and a surface-geometry factor. Since the profile factor remains of order unity for the smooth temperature distributions encountered here, the dominant variation is controlled by the spatial variation of $\beta_{\mathrm{crit}}$. This leads to the geometry term

(23) $$\frac{\nabla_{2D}^{2}\vartheta_{\mathrm{peak}}}{\vartheta_{\mathrm{peak}}}=-\frac{\nabla_{2D}^{2}\beta_{\mathrm{crit}}}{\beta_{\mathrm{crit}}}+2\frac{∥\nabla_{2D}\beta_{\mathrm{crit}}{∥}^{2}}{\beta_{\mathrm{crit}}^{2}},$$

showing that the tendency for lateral heat redistribution is governed not by the absolute curvature itself, but by the spatial variability of the local stability limit. In other words, regions where $\beta_{\mathrm{crit}}$ changes rapidly are precisely the regions where lateral conduction becomes most significant. This provides a direct link between shell geometry and the validity of the locally one-dimensional thermal model, and allows that validity to be assessed from geometric considerations alone.

A further important consequence of the perturbation analysis is that it provides a differentiable semi-analytical representation of $\beta_{\mathrm{crit}}$. This converts the geometric stability limit from a quantity that would otherwise require repeated numerical solution of a boundary-value problem into an explicit function of the local geometry. Consequently, the surface gradient and Laplacian appearing in Equation (23) can be evaluated analytically.

Although Equation (23) establishes a direct connection between the flux ratio and the local geometry, it does not lead to a universally applicable criterion expressed solely in terms of curvature magnitude. The analysis shows that lateral heat redistribution is governed by spatial derivatives of the stability parameter $\beta_{\mathrm{crit}}$, and therefore by curvature gradients and curvature Laplacians rather than by the absolute curvature itself. Consequently, two laminates with comparable principal curvatures may exhibit markedly different flux ratios if the curvatures vary differently over the surface.

This observation is both a limitation and a strength of the present analysis. It implies that a rigorous assessment of the validity of the locally one-dimensional approximation generally requires evaluation of the geometric factor for the specific geometry under consideration. Such an evaluation is entirely feasible when a digital representation of the laminate mid-surface is available, for example from a CAD model, since the required geometric quantities can be computed directly from the surface description. The analysis therefore provides a practical route for geometry-specific verification of the model assumptions.

At the same time, it is desirable to obtain a more general understanding of the typical magnitude of the flux ratio for engineering geometries. To this end, a small set of representative test cases was selected to span a broad region of the admissible curvature space while also probing a realistic range of curvature gradients. Rather than establishing rigorous bounds, these geometries provide a benchmark for assessing the characteristic levels of lateral heat transport that may be expected in doubly curved laminates and thereby offer insight into the practical range of validity of the locally one-dimensional formulation.

The analytical worst-point estimates presented in Appendix C indicate that the geometric contribution to the flux ratio remains small throughout the investigated curvature range. A particularly important special case is the straight cylinder subjected to uniform thermal boundary conditions. Although one principal curvature is finite everywhere, the curvature field itself is spatially uniform, implying a spatially constant stability limit $\beta_{\mathrm{crit}}$. As a consequence, the gradients and Laplacians that drive the analytical flux-ratio expression vanish identically, and the predicted net lateral conductive divergence is exactly zero.

This result has practical significance because cylindrical shells constitute one of the most common classes of composite structures. Within the assumptions of the present analysis, the local runaway criterion therefore remains valid for a straight cylinder regardless of its curvature or wall thickness. More broadly, the cylinder illustrates a fundamental conclusion of the flux-ratio analysis: finite curvature alone does not generate lateral heat redistribution. Instead, lateral conduction is driven by spatial variations in the local stability limit, and thus by variations in the underlying geometry rather than by curvature magnitude itself.

The saddle and toroidal test geometries probe more demanding regions of curvature space where $\beta_{\mathrm{crit}}$ varies continuously over the surface. Even in these cases, the analytical geometry factor remains modest over the investigated range. For the most severe toroidal geometry considered, corresponding to $\left(\sigma_{1},\sigma_{2}\right)=\left(0.667,-0.286\right)$, the conservative worst-point estimate remains below 0.1. Since the actual flux ratio is obtained by multiplication with the through-thickness profile factor 𝒫<1, the corresponding lateral contribution is predicted to remain at only a few percent even in this deliberately severe case.

These results suggest that substantial departures from local one-dimensional behaviour require rather specific geometric circumstances. Large curvatures by themselves are not sufficient. Instead, the most challenging configurations are those for which the stability surface varies rapidly in curvature space, causing correspondingly large gradients and Laplacians of $\beta_{\mathrm{crit}}$.

The quasi-3D simulations provide an independent numerical assessment of these analytical predictions. In these calculations, the shell mid-surface was extruded through the thickness to form frustum-like control volumes, and the local lateral and transverse heat fluxes were evaluated directly from the numerically computed temperature fields. The selected test geometries probe complementary regions of curvature space: the saddle follows the anticlastic diagonal $\sigma_{1}=-\sigma_{2}$, while the torus provides line scans with finite positive $\sigma_{1}$ and varying $\sigma_{2}$.

Overall, the numerical results support the analytical interpretation. Over the range

$$-0.2≲\sigma_{2}≲0.15,$$

the computed flux ratios remain below approximately $4\times 10^{-3}$, indicating that lateral heat transport is negligible compared with transverse heat extraction throughout most of the investigated domain. Only when the curvature state moves deeper into the anticlastic region does the ratio increase appreciably, reaching the one-percent level at approximately

$$\sigma_{2}\approx -0.25.$$

This behaviour is consistent with the geometry-based interpretation of the analytical model. Anticlastic regions are associated with lower stability margins, larger local temperature overshoots, and therefore steeper lateral temperature gradients. The resulting increase in lateral conductive transport is precisely the trend predicted by the spatial variation of $\beta_{\mathrm{crit}}$.

A more direct comparison can be made by evaluating the analytical flux-ratio estimate at curvature levels corresponding to the quasi-3D simulations. For the toroidal cases with $\sigma_{2}=-0.143$ and $\sigma_{2}=-0.286$, the analytical geometry factors in Table A1 are $G_{\beta }=0.0049$ and $G_{\beta }=0.084$, respectively. Using a representative profile factor of 𝒫≈0.5 gives estimated flux ratios of approximately $2.5\times 10^{-3}$ and $4.2\times 10^{-2}$. Interpolation of the collapsed quasi-three-dimensional trend at the same curvature levels gives corresponding values of approximately $1.0\times 10^{-3}$ and $1.5\times 10^{-2}$.

Although the agreement is not exact, the two approaches consistently identify the same region of curvature space as the limiting case and predict flux ratios of the same order of magnitude. Given that the analytical values are conservative worst-point estimates whereas the numerical values are extracted from finite surface scans, agreement within roughly a factor of three is considered satisfactory. The comparison therefore provides a strong consistency check on the underlying geometry-based formulation.

The practical significance of these findings becomes clearer when viewed through a local Monge-patch analysis. In differential geometry, a Monge patch is a local representation of a smooth surface as a height function above its tangent plane. The approach may be viewed as a systematic “zooming-in” procedure: in a sufficiently small neighbourhood, the surface geometry can be expressed as a polynomial expansion whose coefficients are directly related to the local curvatures and their spatial derivatives. Substituting this local representation into the analytical flux-ratio expression makes it possible to determine how the lateral-conduction correction scales with laminate thickness and geometric length scale. The analysis therefore complements the numerical examples by revealing the asymptotic structure of the problem and identifying the geometric mechanisms responsible for the flux-ratio behaviour.

The resulting scaling analysis shows that the lateral-conduction correction decreases at least cubically with the thickness-to-radius ratio

$$\epsilon =\frac{w}{R},$$

where *w* is the laminate half-thickness and *R* should be interpreted as the characteristic length scale over which the curvature field varies appreciably, not merely as the smallest local principal radius of curvature. In order-of-magnitude form,

(24) $$R_{\mathrm{flux}}∼𝒪\left(1\right)\left[\frac{\epsilon^{3}+\epsilon^{4}}{\beta_{\mathrm{crit}}}+\frac{\epsilon^{4}}{\beta_{\mathrm{crit}}^{2}}\right],$$

where the order-unity prefactor includes the profile factor and, for anisotropic materials, the conductivity ratio. This result provides a theoretical explanation for the small flux ratios observed in both the analytical and quasi-3D studies. For slender laminates, lateral conductive transport diminishes much more rapidly than transverse conduction as the thickness-to-radius ratio decreases. The scaling therefore explains why the locally one-dimensional approximation remains accurate over a surprisingly broad portion of curvature space.

Equally importantly, the scaling identifies the situations most likely to challenge the approximation. The leading corrections become largest where $\beta_{\mathrm{crit}}$ varies most rapidly, which is precisely the behaviour observed in the anticlastic regions highlighted by both the analytical estimates and the quasi-3D simulations.

Taken together, the analytical flux-ratio estimates, the quasi-3D simulations, and the Monge-patch scaling analysis all support the same conclusion. The validity of the locally one-dimensional approximation is controlled primarily by geometric variation rather than by curvature magnitude alone. Smooth geometries with moderate curvature are comfortably within the validity range, while the most demanding cases occur in strongly anticlastic regions where the stability surface varies rapidly in curvature space.

For the geometries examined here, flux ratios remain below approximately one percent throughout most of the investigated domain. The saddle and toroidal simulations indicate that anticlastic states with $|\sigma_{1}\left|\;\approx \;\right|\sigma_{2}|\;≲\;0.25$ remain well described by the locally one-dimensional formulation, whereas the toroidal scans demonstrate that substantially larger positive curvature components can be tolerated near cylindrical states. For geometries lying outside these representative ranges, or for cases involving strongly asymmetric thermal boundary conditions, the geometry-based flux-ratio criterion provides a straightforward means of performing a geometry-specific validity assessment directly from the surface description.

## 4. Discussion

The power of dimensional reduction and universal scaling. Although modern computational power enables direct numerical simulation of thermal runaway in specific geometries, such point solutions provide limited insight into the governing physics. By reducing the problem dimensionally, the high-dimensional space of physical variables collapses into a small set of governing parameters $\left(Da,A_{r},Bi,\sigma \right)$. For the local stability criterion, curvature and thickness enter through the dimensionless curvature σ=κw, while chemistry and processing temperature enter through the Arrhenius scaling. This separation exposes scaling laws that are otherwise obscured and makes it possible to construct a transparent safe-processing criterion. The separate flux-ratio analysis then provides a consistency check on the locally one-dimensional reduction by assessing whether lateral conductive divergence remains small compared with through-thickness conduction.

**Validity and generality across curved shell geometries.** The dimensional reduction used in this work treats a curved laminate as a collection of local through-thickness thermal elements. Once lateral heat transport is negligible, the stability margin at each surface point is governed by the local principal curvatures, the local boundary heat-transfer conditions, and the material/process parameters entering the Damköhler criterion. This provides a practical route for applying the theory to general shell geometries: the principal curvatures can be evaluated pointwise from either an analytical surface description or a digital surface representation, such as a CAD model, and the local value of $\beta_{\mathrm{crit}}$ can then be computed directly.

The location controlling the runaway criterion is the point where $\beta_{\mathrm{crit}}$ is smallest, since this gives the lowest local critical Damköhler number. This search is substantially simpler than a full assessment of lateral heat transport, because it only requires local curvature and boundary-condition information. The flux-ratio analysis addresses a different question: whether the lateral conductive term omitted in the locally one-dimensional reduction remains small. That check depends not only on the local curvature state, but also on how the curvature field varies over the surface, through the gradients and Laplacian of $\beta_{\mathrm{crit}}$.

The analytical flux-ratio estimate and the quasi-three-dimensional simulations show that the locally one-dimensional approximation is robust for the representative smooth shell geometries investigated here. The computed flux ratios remain well below one percent over most of the explored curvature range, with the largest values occurring in strongly anticlastic regions where the stability function varies most rapidly. Straight cylindrical shells with uniform boundary conditions constitute an important limiting case: although one principal curvature is finite, the curvature field is spatially constant and the net lateral conductive divergence vanishes identically. Thus, the model is not limited by curvature magnitude alone, but by the spatial variation of the geometry-dependent stability factor.

For most practical process-design tasks, it is therefore reasonable to first apply the local stability criterion by identifying the minimum value of $\beta_{\mathrm{crit}}$ over the surface. If the resulting design margin is large, and the part geometry is smooth, a separate flux-ratio evaluation may not be necessary. If the design margin is narrow, if the geometry contains rapidly varying anticlastic regions, or if experiments are costly compared with additional analysis, the geometry-based flux-ratio criterion in Appendix C provides a direct way to verify the locally one-dimensional assumption.

**Role of the fitting coefficients in the reduced order model** The fitted coefficients used in the reduced-order model should be interpreted as low-order surrogate representations of numerically precomputed stability data, rather than as independent empirical parameters. The differentiability of this surrogate representation is also what makes the geometry-based flux-ratio estimate possible, since the required gradients and Laplacians of $\beta_{\mathrm{crit}}$ can be evaluated directly. The intended use of the semi-analytical solution is interpolation within the calibration domain considered in the present study; extrapolation beyond this range should be treated with caution. Within the fitted domain, the residual fitting errors are small and are expected to contribute less to the overall prediction uncertainty than the larger uncertainties typically associated with cure kinetics, thermal properties, and heat-transfer boundary conditions.

**Implications of the kinetic approximation.** The perturbation analysis is based on a simplified representation of cure kinetics in which the reaction-rate shape factor is replaced by an effective constant $C_{\alpha }$. This approximation provides a conservative estimate of heat generation, since it neglects the reduction in reaction rate associated with reactant depletion at higher degrees of cure. As a result, the predicted stability limits systematically err on the safe side.

The sensitivity analysis across different kinetic exponents (m,n) shows that the approximation remains robust over a wide range of practically relevant curing behaviours. At the same time, the results demonstrate that relaxing the assumption $C_{\alpha }=\max f\left(\alpha \right)$ leads to improved agreement with the fully coupled numerical solution. In practice, this introduces a clear trade-off: using the maximum value yields a strictly conservative criterion without calibration, while treating $C_{\alpha }$ as an effective parameter allows higher predictive accuracy.

The predictive accuracy of the present framework for any specific resin system remains conditional on the quality of the underlying cure-kinetics description. Thermal runaway predictions are inherently sensitive to kinetic parameters because the reaction source term depends both exponentially on temperature and nonlinearly on degree of cure. The proposed formulation does not eliminate this sensitivity; rather, it separates the chemistry-dependent contribution from the geometric and heat-transfer stability factor, thereby making the respective roles of kinetics, heat transfer and geometry more transparent. In practical applications, uncertainties in cure kinetics are therefore expected to influence the predicted critical conditions at least as strongly as the residual error associated with the reduced-order geometric approximation. The present model should consequently be viewed as an engineering-oriented reduced-order tool whose practical reliability depends on the availability of well-characterized kinetic input data.

**Influence of material property variations.** The present analysis assumes constant thermo-physical properties, evaluated at representative curing conditions. In practice, properties such as thermal conductivity, heat capacity, and reaction enthalpy may vary with temperature and degree of cure. These variations can influence both heat generation and heat diffusion, potentially modifying the stability boundary.

However, for typical thermoset systems, these variations are moderate over the relevant temperature range and primarily affect the quantitative value of the stability limit rather than its overall structure. The dimensionless formulation introduced here remains valid, with property variations effectively altering the numerical values of the governing parameters. For higher-fidelity predictions, temperature-dependent properties can be incorporated in the numerical calibration stage without modifying the semi-analytical framework.

In materials with strong conductivity anisotropy, the stability criterion based on through-thickness conduction remains applicable through the transverse conductivity, while the flux-ratio validity check should include the conductivity ratio $\Xi_{k}=\lambda_{\|}/\lambda_{⊥}$.

**Neglected effects and model scope.** Certain physical effects are not included in the present formulation. Radiative heat transfer is neglected in favour of convective (Robin) boundary conditions. For the moderate temperatures typical of composite curing, radiative contributions are generally small compared to convection and conduction, but may become relevant at elevated temperatures or in high-temperature processing environments.

The model also does not account for cure-induced shrinkage or the development of residual stresses. These phenomena primarily influence mechanical integrity rather than the thermal stability limit itself, although strong coupling between deformation and heat transfer could have a secondary effect in specific cases. Incorporating such effects would require a coupled thermo-mechanical formulation, which is beyond the scope of the present study.

These simplifications are consistent with the goal of developing a reduced-order stability model and do not affect the primary conclusions regarding thermal runaway behaviour.

**Interpretation of model accuracy and engineering use.** The semi-analytical framework developed in this work is intended as a reduced-order predictive tool for process design and stability assessment, rather than as a high-precision numerical model. Its primary strength lies in capturing the correct scaling, trends, and relative sensitivity of the thermal-runaway limit with respect to geometry, heat-transfer conditions, and processing temperature.

The reported deviations (on the order of 10–20% for the critical Damköhler number) should be interpreted in engineering terms. Because the Damköhler number scales with the square of the characteristic laminate thickness, an average error of approximately 20% in $Da_{\mathrm{crit}}$ corresponds to only about 9–10% in the predicted safe thickness limit. Likewise, because $Da_{\mathrm{crit}}$ depends exponentially on the processing temperature through the Arrhenius term, the same error corresponds to only a modest shift in the predicted critical processing temperature. For the representative values used in the present study ($A_{r}\approx 32.4$ and $T_{\infty }\approx 2.0$), linearization of the temperature dependence gives $\Delta T_{\infty }\approx 0.025$, i.e., about 1.3% in dimensionless temperature. These estimates place the reduced-order model error in a more practical context and support its use as an engineering-oriented screening tool, while still recognizing that detailed process qualification requires material-specific calibration and, where needed, higher-fidelity simulation.

**Robustness under Asymmetric Boundary Conditions.** Perfectly symmetric cooling is rare in practice, which makes asymmetric boundary conditions essential to consider. The results show that stability is strongly influenced by the contrast between the two boundary heat-transfer conditions and by the orientation of curvature relative to the better- and worse-cooled surfaces. This insight has direct practical implications. For example, improved convection on the accessible surface can compensate for limited tool-side heat extraction, an effect that can be quantified directly using the semi-analytical model without resorting to full 3D simulations.

**Practical use of the stability criterion.** The proposed framework is designed to be directly applicable in engineering process design. Its use is straightforward when the surface geometry is known, either from an analytical description or from a CAD model. The local principal curvatures are extracted from the mid-surface, converted to dimensionless form through $\sigma_{i}=\kappa_{i}w$, and used to evaluate the local stability factor $\beta_{\mathrm{crit}}$. The most critical point for thermal runaway is then identified by the minimum value of $\beta_{\mathrm{crit}}$, or equivalently by the minimum value of $Da_{\mathrm{crit}}$, over the surface.

A typical workflow is:

1. Extract local principal curvatures $\left(\kappa_{1},\kappa_{2}\right)$ from the part geometry and compute the dimensionless curvatures $\sigma_{i}=\kappa_{i}w$.
2. Estimate the effective Biot numbers $\left(Bi_{d},Bi_{u}\right)$ from the expected surface heat-transfer conditions.
3. Evaluate the geometric stability factor $\beta_{\mathrm{crit}}\left(\sigma_{1},\sigma_{2},Bi_{d},Bi_{u}\right)$ using the semi-analytical expressions.
4. Choose the process temperature, initially for example the manufacturer’s recommended cure temperature.
5. Compute $Da_{\mathrm{crit}}$ from Equation (21).
6. Compare the process Damköhler number Da with $Da_{\mathrm{crit}}$. The minimum value of $Da_{\mathrm{crit}}$ over the surface controls the overall process safety.
7. If $Da>Da_{\mathrm{crit}}$, stabilise the cure by improving heat transfer and/or reducing the processing temperature until $Da\leq Da_{\mathrm{crit}}$. The part should then be held under these stable conditions during an initial dwell step, so that enough reactants are consumed before the temperature is raised to the recommended post-cure level.

If the geometry contains rapidly varying curvature, strongly anticlastic regions, or strongly asymmetric boundary conditions, the flux-ratio criterion in Appendix C can be evaluated as an additional consistency check. This requires more geometrical information, because gradients and Laplacians of the stability factor (see Appendix C) must be estimated over the surface. In many engineering situations this additional cost is small compared with experimental trial-and-error, especially when the geometry is already available digitally.

As a compact worked example, consider the saddle-shaped shell in Figure 1 with a horizontal width of 1 m and a total thickness of 30 mm (w=15 mm). We will assume that the heat-transfer conditions are symmetric with Bi=10 corresponding to weak convective cooling and that the material properties and recommended processing conditions are the same as in [4], which was shown in Section 3.2 to yield dimensionless parameters $T_{\infty }=2.33$, $A_{r}=32.4$, m=0.313, n=1.66, and a process Damköhler number $Da=3.4\times 10^{6}$. All data and semi-analytical formulas needed to compute the critical Damköhler number were implemented in a standard spreadsheet program to simplify parameter sweeps.

The geometric stability factor $\beta_{\mathrm{crit}}$ is evaluated using Equation (12) and the curvatures from the geometrical model that was used to generate Figure 1. The lowest value for $\beta_{\mathrm{crit}}$ is found at the centre of the saddle where $\left(\sigma_{1},\sigma_{2}\right)=\left(0.1,-0.1\right)$, which yields $\beta_{\mathrm{crit}}=0.72$. The resulting critical Damköhler number is $Da_{\mathrm{crit}}=3.12\times 10^{5}$, which is more than one order of magnitude lower than the process value $Da=3.4\times 10^{6}$. This indicates that the cure cycle will lead to thermal runaway.

To achieve a stable cure cycle, the process temperature is reduced until the critical Damköhler number equals the process Damköhler number. A simple parameter sweep shows that the process temperature needs to be lowered to $T_{\infty }=1.945$ to reach the criticality limit. For this particular material (using data from [4]) this corresponds to a physical cure temperature of 116 °C, and the predicted peak overshoot (at the centre of the saddle) is $T_{\max,\mathrm{crit}}=0.153$ (30.7 °C).

If the heat-transfer conditions can be improved to Bi=1000 (strong forced convection), the process temperature can be increased to $T_{\infty }=1.97$ (121 °C), while the predicted peak temperature overshoot increases by only 0.7 °C.

The need to reduce the process temperature in order to avoid thermal runaway naturally leads to multi-stage cure cycles. In such cases, a dwell period at a reduced temperature is typically followed by a post-cure at the manufacturer’s recommended temperature to ensure full conversion. While the present formulation does not explicitly predict the required dwell time, useful estimates can be obtained from the same stability analysis.

In dimensional terms, the duration of the exothermic peak is generally limited to a few thermal diffusion time units $\tau =w^{2}\rho c_{p}/\lambda$, with the initial heat-up occurring over roughly one time unit. Since the peak temperature corresponds closely to the maximum of the reaction rate function f(α), a decay in temperature after the peak indicates that the reaction rate has started to decrease due to conversion. This suggests that a dwell time on the order of 3–4 thermal diffusion time units is sufficient in most cases to safely pass the critical stage before increasing the temperature.

Alternatively, in practical processing, the dwell time can be determined by monitoring the temperature evolution. The time at which the peak temperature is reached, augmented with a suitable safety margin, provides a robust indicator that the most critical part of the cure has been completed. These simple strategies allow multi-stage cure cycles to be designed without requiring full transient simulations, while remaining consistent with the stability framework developed in this work.

This example illustrates the main practical advantage of the formulation: once the local curvature field and boundary heat-transfer conditions are known, the critical location and safe process window can be estimated without performing a full transient three-dimensional simulation. If desired, the same geometry can subsequently be used for the more detailed flux-ratio check described in Appendix C.

**Advantages of the semi-analytical formulation.** As discussed in Section 3.2, the numerical evaluation of the stability limit is computationally inexpensive, with both boundary-value solutions and transient simulations requiring only modest computational effort. The motivation for the present semi-analytical formulation is therefore not primarily to reduce computational cost.

Instead, the main advantage lies in providing a direct, differentiable, and physically transparent representation of the stability limit. The fitted expressions should be interpreted as compact interpolating representations of the numerically resolved stability surface, rather than as closed-form analytical solutions of the underlying boundary-value problem. Within their calibration range, they make the dependence of $\beta_{\mathrm{crit}}$ on curvature and boundary heat transfer explicit, while preserving the fundamental symmetries of the governing problem.

This explicit differentiable representation is also essential for the analytical flux-ratio analysis. The flux-ratio criterion requires surface gradients and Laplacians of $\beta_{\mathrm{crit}}\left(\sigma_{1},\sigma_{2}\right)$. Without a smooth semi-analytical representation of the stability surface, these derivatives would have to be estimated by repeated numerical solution of the boundary-value problem followed by numerical differentiation, which would obscure the structure of the criterion and make the geometry-based validity assessment much less transparent.

In addition, the semi-analytical expressions can be implemented in simple environments such as spreadsheets, enabling rapid evaluation without specialised numerical tools. This ease of use is particularly valuable in engineering practice, where many configurations must be assessed and where quick, reliable estimates of stability limits are often required.

**Consistency Between Dynamic Detection and Perturbation Theory.** The second-derivative criterion aligns closely with the perturbation-based stability limit. In the perturbation framework, runaway occurs when no steady temperature profile can balance heat generation and conduction. Dynamically, this same transition is detected when ${\ddot{T}}_{s}=0$, marking the onset of self-acceleration. The agreement between these independent approaches shows that the transient acceleration signal reflects the same underlying stability limit as the semi-analytical theory. This provides strong support for using the second derivative as an early-warning indicator in practical applications.

**Real-Time Monitoring and Active Process Control.** The second-derivative criterion used to classify the numerical solutions also provides a practical basis for real-time process monitoring. During stable curing, heat removal dominates and the temperature response is decelerating $\left({\ddot{T}}_{s}<0\right)$. As ${\ddot{T}}_{s}$ increases and approaches zero from the negative side, the system reaches a critical balance where heat generation begins to exceed the cooling capacity. This transition occurs before large temperature rises develop, providing an opportunity for early detection of impending thermal runaway.

Monitoring the curvature of the temperature signal therefore offers a simple and physically grounded early-warning indicator, enabling timely intervention, for example by reducing the tool temperature and introducing a temporary dwell. In this way, the tooling can act as an active control element rather than merely a passive thermal boundary.

In experimental applications, the evaluation of the second derivative requires differentiation of measured temperature signals, which may be affected by measurement noise. Appropriate signal conditioning is therefore necessary to obtain a stable estimate of the temperature acceleration. Simple smoothing techniques, such as moving averages or Savitzky–Golay filtering, which preserve the local curvature of the signal while reducing high-frequency noise, can be applied prior to differentiation. This allows the second-derivative criterion to be implemented as a robust and practical early-warning tool in real processing environments.

## 5. Conclusions

In this work, a semi-analytical stability framework for thermal runaway during the cure of thick composite shells has been developed and validated. The main conclusions are:

- **A unified stability criterion for curved composite shells.** A local runaway criterion has been derived for curved shell geometries within the scope of the locally one-dimensional through-thickness formulation and convective (Robin) boundary conditions. The resulting expression for the critical Damköhler number, $Da_{\mathrm{crit}}$, separates geometry and boundary heat transfer from reaction sensitivity and processing temperature. This provides a transparent stability margin, $Da/Da_{\mathrm{crit}}$, that can be evaluated pointwise over a curved laminate surface.
- **Geometry and chemistry separate cleanly.** The critical stability factor $\beta_{\mathrm{crit}}$ contains the influence of local principal curvatures and Biot numbers, whereas the chemical and thermal sensitivity enter through the Arrhenius scaling and the effective kinetic factor $C_{\alpha }$. Validation against fully coupled transient simulations confirms that this separation captures the dominant behaviour over the investigated range of curvatures, boundary conditions, temperatures, and kinetic exponents.
- **The locally one-dimensional approximation is supported by a flux-ratio analysis.** The assumption that through-thickness conduction dominates over lateral conduction was assessed using both an analytical flux-ratio criterion and quasi-three-dimensional numerical simulations. The analytical result shows that the relevant lateral contribution is governed by spatial derivatives of the geometry-dependent stability factor $\beta_{\mathrm{crit}}$, not by curvature magnitude alone. For smooth shell geometries, the lateral correction decreases rapidly with thickness-to-geometric-length ratio, and the quasi-three-dimensional simulations confirm that the flux ratio remains small over the representative curvature range investigated. The most restrictive cases are strongly anticlastic regions where $\beta_{\mathrm{crit}}$ varies rapidly over the surface.
- **Straight cylindrical shells form an important exact limiting case.** For straight cylinders with uniform thermal boundary conditions, the curvature field is spatially constant. The gradients and Laplacians that drive the analytical flux-ratio expression therefore vanish, giving zero net lateral conductive divergence within the assumptions of the analysis. This supports the use of the local runaway criterion for a broad and practically important class of cylindrical composite components.
- **The semi-analytical representation is essential for practical evaluation.** Although the underlying stability surface is obtained from numerical solutions of a non-linear boundary-value problem, its compact semi-analytical representation makes the criterion directly usable. It enables rapid evaluation of $\beta_{\mathrm{crit}}$, preserves the relevant geometric symmetries, and provides the differentiable form required for the analytical flux-ratio criterion. The resulting expressions can be implemented in simple tools such as spreadsheets or applied directly to curvature data extracted from CAD models.
- **Runaway behaviour depends strongly on Arrhenius sensitivity.** The transient simulations show that low Arrhenius numbers lead to gradual, mild transitions, whereas high Arrhenius numbers produce ignition-like behaviour with long induction periods followed by rapid temperature rise. The semi-analytical framework captures the corresponding stability limit and provides a way to distinguish process conditions where deviations are likely to be manageable from those where small changes can trigger severe runaway.
- **Boundary conditions and part orientation matter.** The Biot numbers strongly influence the stability margin, and asymmetric thermal boundary conditions introduce a directional sensitivity to curvature. The same shell geometry may therefore have different stability limits depending on its orientation relative to the better- and worse-cooled surfaces. This provides a direct way to quantify the stabilising effect of improved heat transfer or modified tooling conditions.
- **The framework is directly applicable to engineering process design.** Once the laminate mid-surface geometry and boundary heat-transfer conditions are known, the critical location can be identified by evaluating $Da_{\mathrm{crit}}$ over the surface. This enables rapid estimation of safe thickness limits, identification of critical regions, and assessment of process modifications such as reduced dwell temperatures or improved cooling. If needed, the same geometric data can be used for the more detailed flux-ratio check to verify the locally one-dimensional assumption.

Together, these results establish a physically transparent and practically useful reduced-order framework for predicting and preventing thermal runaway in thick curved composite structures.

## Figures and Tables

**Table 1:** Optimised coefficients for the asymmetric heating case with $Bi_{u}=1000$ and $Bi_{d}$ variable.

Parameter Name	Coeff 1	Coeff 2	Coeff 3
Global stability $c_{0}$	$c_{1}=0.88534$	$c_{2}=0.22$	$c_{3}=1.54625$
Thermal tilt *L*	$L_{1}=0.19013$	$L_{2}=1.29248$	-
Mean curvature *a*	$a_{1}=-0.11991$	$a_{2}=0.16400$	$a_{3}=1.54028$
Gaussian curvature *b*	$b_{1}=0.42387$	$b_{2}=0.40639$	$b_{3}=1.30863$

**Table 2:** Asymmetric heating with a nearly isothermal outer surface ($Bi_{u}=1000$): single-parameter calibration results over the curvature grid $\left(\sigma_{1},\sigma_{2}\right)$. Errors are relative to numerical $Da_{\mathrm{crit}}$.

${\mathrm{Bi}}_{d}$	$C_{\alpha }$	RMS Error (%)	Max Error (%)
0	0.213	11.56	55.86
1	0.220	3.30	6.45
3	0.212	2.34	6.48
10	0.213	1.90	4.69
100	0.215	1.76	4.65

**Table 3:** Calibration sweep over the kinetic exponents (m,n). For each pair, a full chemical calibration over $\left(A_{r},T_{\infty }\right)$ was performed. The table reports the fitted chemical prefactor $C_{\alpha }$, the relative error of $Da_{\mathrm{crit}}$, the normalised damping factor $K=C_{\alpha }/f_{\max}$, the fitted temperature scaling $C_{T}$, and the relative error of $\Delta T_{\max}$.

*m*	*n*	$C_{\alpha }$	Error (${\mathrm{Da}}_{\mathrm{crit}}$) [%]	$C_{T}$	Error ($\Delta T_{\max}$) [%]	*K*
0.30	1.00	0.345	15.1	1.351	4.6	0.6968
0.30	1.18	0.315	16.8	1.355	5.7	0.6629
0.30	1.35	0.289	18.4	1.352	7.3	0.6315
0.30	1.52	0.266	20.0	1.343	9.1	0.6022
0.30	1.70	0.247	21.6	1.331	10.8	0.5747
0.35	1.00	0.345	13.1	1.238	4.6	0.7475
0.35	1.18	0.315	14.7	1.251	4.7	0.7154
0.35	1.35	0.289	16.2	1.256	5.3	0.6852
0.35	1.52	0.266	17.6	1.254	6.3	0.6565
0.35	1.70	0.247	19.0	1.249	7.7	0.6294
0.40	1.00	0.348	11.1	1.112	6.5	0.8049
0.40	1.18	0.317	12.7	1.133	5.5	0.7740
0.40	1.35	0.291	14.2	1.146	5.2	0.7447
0.40	1.52	0.268	15.5	1.153	5.3	0.7169
0.40	1.70	0.248	16.9	1.155	5.8	0.6906
0.45	1.00	0.358	9.6	0.967	11.2	0.8797
0.45	1.18	0.324	11.0	1.000	9.2	0.8465
0.45	1.35	0.296	12.3	1.023	7.5	0.8148
0.45	1.52	0.272	13.5	1.038	6.4	0.7861
0.45	1.70	0.252	14.7	1.048	5.8	0.7591
0.50	1.00	0.374	9.3	0.809	14.0	0.9723
0.50	1.18	0.338	10.2	0.849	13.0	0.9395
0.50	1.35	0.308	11.0	0.880	11.7	0.9075
0.50	1.52	0.283	12.1	0.905	10.3	0.8772
0.50	1.70	0.261	13.3	0.924	9.2	0.8482

**Table 4:** Global model validation using an 8-run fractional factorial design. Each case represents a full $\left(A_{r},T_{\infty }\right)$ grid calibration. The global model predictions for $C_{\alpha }$ and $C_{T}$ are compared against the direct numerical calibration to determine the relative error in $Da_{\mathrm{crit}}$ and $\Delta T_{\max}$. Symmetric cooling implies $Bi_{d}=Bi_{u}=1000$, while Asymmetric implies $Bi_{d}=10,Bi_{u}=1000$.

		Parameter Set				$C_{\alpha }$ (for ${\mathrm{Da}}_{\mathrm{crit}}$)			$C_{T}$ (for $\Delta T_{\max}$)		
Case	Geometry	Heating	$\left(\sigma_{1},\sigma_{2}\right)$	m	n	Model	Num.	Error [%]	Model	Num.	Error [%]
1	Flat Plate	Sym.	(+0.0,+0.0)	0.3	1.0	0.3440	0.3452	0.35	1.3507	1.3508	0.00
2	Sphere (+)	Sym.	(+0.4,+0.4)	0.5	1.7	0.2605	0.2616	0.42	0.9237	0.9304	0.72
3	Sphere (−)	Sym.	(−0.4,−0.4)	0.3	1.7	0.2473	0.2481	0.34	1.3281	1.3423	1.06
4	Saddle	Sym.	(+0.4,−0.4)	0.5	1.0	0.3726	0.3666	1.64	0.8104	0.8047	0.71
5	Flat Plate	Asym.	(0.0,−0.0)	0.5	1.7	0.2605	0.2589	0.60	0.9237	0.9229	0.09
6	Sphere (+)	Asym.	(+0.4,+0.4)	0.3	1.0	0.3440	0.3396	1.28	1.3507	1.3775	1.95
7	Sphere (−)	Asym.	(−0.4,−0.4)	0.5	1.0	0.3726	0.3704	0.61	0.8104	0.8188	1.03
8	Saddle	Asym.	(+0.4,−0.4)	0.3	1.7	0.2473	0.2418	2.25	1.3281	1.3152	0.98

**Figure A1:**
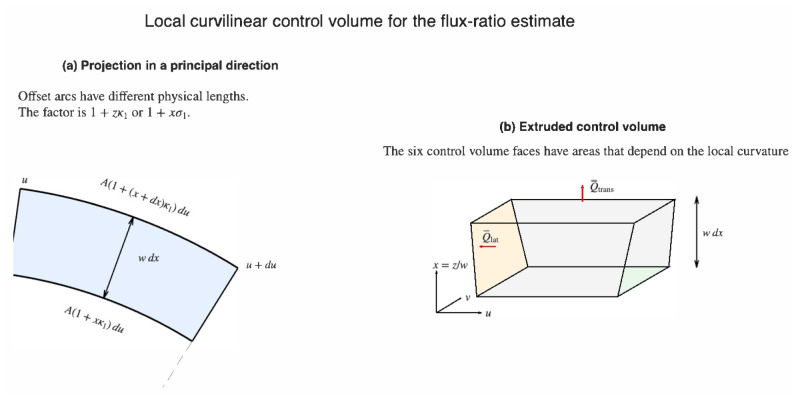
Local curvilinear control volume used in the flux-ratio estimate. (**a**) Projection in one principal direction. Offset surfaces have different physical arc lengths because the radius changes through the thickness, giving the factor $1+z\kappa_{i}=1+x\sigma_{i}$. (**b**) The full control volume is obtained by combining the two principal directions and the through-thickness direction. The six bounding faces are used to compare the net lateral conductive divergence with the transverse conductive contribution.

**Figure A2:**
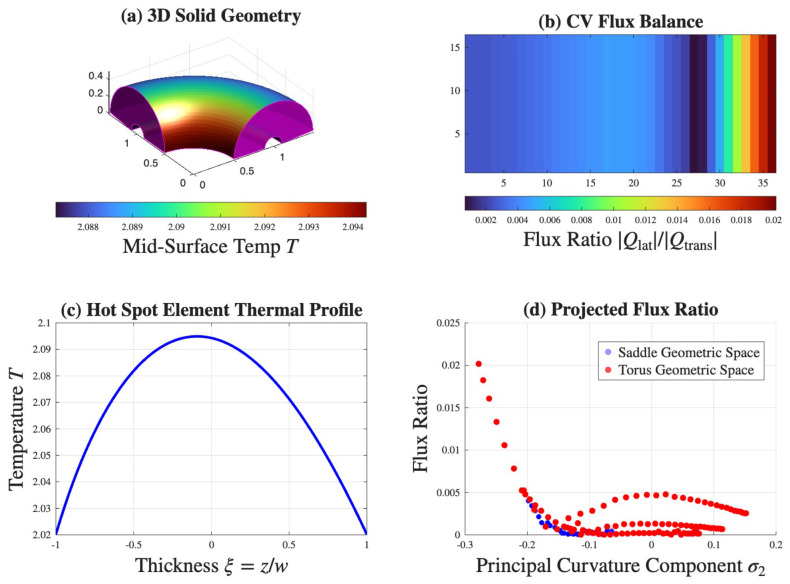
Quasi-three-dimensional assessment of the locally one-dimensional approximation. (**a**) Extruded shell geometry for the quarter-torus with H=0.4. (**b**) Corresponding surface distribution of the local flux ratio $R_{\mathrm{flux}}$. (**c**) Through-thickness temperature profile in the hottest thermal element at its peak time. (**d**) Flux ratio plotted against $\sigma_{2}$ for the combined saddle and toroidal simulations. The saddle data follow the anticlastic line $\sigma_{1}=-\sigma_{2}$, while the toroidal data correspond to line scans with finite positive $\sigma_{1}$.

## Data Availability

The MATLAB source code for all programs used to generate the results and figures is provided in the Supplementary Material and is free to use under the GNU General Public License.

## Supplementary Materials

The following supporting information can be downloaded at: https://www.mdpi.com/article/10.3390/ma19143132/s1, The supplementary material contains the MATLAB source code, data, and documentation files required to reproduce all numerical results and figures reported in this study.

## Abbreviations and Main Variables

Symbol/Abbreviation	Meaning
Abbreviations	
BVP	Boundary Value Problem
DNS	Direct Numerical Simulation
FRP	Fibre-Reinforced Polymer
FVM	Finite Volume Method
ODE	Ordinary Differential Equation
PDE	Partial Differential Equation
3D	Three-Dimensional
2D	Two-Dimensional
Thermal properties and physical parameters	
ρ	Density [$\mathrm{kg}\;m^{-3}$]
$c_{p}$	Specific heat capacity [$J\;{\mathrm{kg}}^{-1}\;K^{-1}$]
λ	Thermal conductivity (through-thickness) [$W\;m^{-1}\;K^{-1}$]
$q_{c}$	Heat of reaction per unit mass [$J\;{\mathrm{kg}}^{-1}$]
*A*	Pre-exponential factor [$s^{-1}$]
*E*	Activation energy [$J\;{\mathrm{mol}}^{-1}$]
*R*	Universal gas constant [$J\;{\mathrm{mol}}^{-1}\;K^{-1}$]
$h_{\mathrm{eff}}$	Effective heat-transfer coefficient [$W\;m^{-2}\;K^{-1}$]
Key variables	
*T*	Temperature
α	Degree of cure
$T_{\infty }$	Reference (boundary) temperature
*w*	Half-thickness of the laminate
$\kappa_{1},\kappa_{2}$	Principal curvatures
$\sigma_{1},\sigma_{2}$	Dimensionless curvatures $\left(\kappa_{i}w\right)$
Dimensionless parameters and model constants	
Da	Damköhler number (reaction-to-diffusion ratio)
$A_{r}$	Arrhenius number (temperature sensitivity)
$Bi_{u,d}$	Biot numbers (boundary heat-transfer strength)
β	Stability parameter $\beta =Da\;C_{\alpha }\exp\left(-\frac{A_{r}}{T_{\infty }}\right)\frac{1}{\varepsilon }$
$\beta_{\mathrm{crit}}$	Critical stability factor (geometry + boundary effects)
$C_{\alpha }$	Effective kinetic factor approximating f(α)
$\Delta T_{\mathrm{adi}}$	Adiabatic temperature rise
ε	Perturbation scaling parameter $\left(T_{\infty }^{2}/A_{r}\right)$
$\Xi_{k}$	Thermal-conductivity anisotropy ratio $\left(\lambda_{\|}/\lambda_{⊥}\right)$
Note: In the analysis, dimensional variables are reused to denote their dimensionless counterparts after normalisation where no ambiguity arises. A tilde notation (e.g., $\tilde{T},\tilde{x}$) is introduced temporarily during the non-dimensionalisation procedure (Section 2.2), but is dropped in subsequent derivations for clarity.	

## Appendix A. Derivation of the Perturbation Stability Criterion

This appendix provides a concise derivation of the perturbation-based stability criterion, starting from the dimensionless energy and kinetic equations (Equations (4) and (5)).

### Appendix A.1. Controlled Approximations

To transform the transient coupled system into a tractable stability problem, three approximations are introduced:

1. **Decoupling of kinetics**
  The autocatalytic factor f(α) is replaced by its maximum value $C_{\alpha }=f\left(\alpha_{\max}\right)$, where $\alpha_{\max}=m/\left(m+n\right)$. This produces a conservative, worst-case estimate of heat generation.
  With this approximation, the kinetic rate expression in Equation (5) becomes independent of α, allowing ∂α/∂t in the energy equation to be replaced directly by its right-hand side. The heat-generation term therefore becomes a function of temperature only, and the coupled thermo-kinetic system reduces to a single equation for *T*.
2. **Small temperature-rise expansion**
  The temperature field is expanded as a perturbation series about the zeroth-order solution $T_{0}$:
  (A1) $$T\left(x,t\right)=T_{0}\left(x,t\right)+\varepsilon \vartheta \left(x,t\right)+𝒪\left(\varepsilon^{2}\right)$$
  where ϑ is the first-order perturbation function. The dimensionless small parameter ε will be selected later to simplify the resulting perturbation equation.
3. **Steady-state limit**
  For the original coupled thermo-kinetic system (Equations (4) and (5)), a true steady state does not exist because the chemical reaction progressively depletes the reactants. However, following the decoupling approximation above, the heat-generation term becomes independent of α, and the system admits a stationary solution in which heat generation and heat removal can balance.
  From a stability perspective, this steady-state limit corresponds to the most critical condition: if the system can sustain a balance between heat release and heat removal at the highest accessible temperature level, it will remain stable throughout the preceding transient. Conversely, loss of a steady solution signals the onset of thermal runaway. The analysis is therefore formulated in the steady-state limit, where ∂T/∂t=0.
  The perturbation ansatz becomes
  $$T\left(x\right)=T_{\infty }+\varepsilon \vartheta \left(x\right)+𝒪\left(\varepsilon^{2}\right),$$
  where the zeroth-order solution has been replaced by its stationary value $T_{\infty }$.

### Appendix A.2. The First-Order Perturbation Equation

To capture the narrow transition regime leading to thermal runaway, we employ high activation energy asymptotics [14] by introducing the small parameter $\varepsilon =T_{\infty }^{2}/A_{r}\ll 1$. This scaling can be understood directly from the structure of the Arrhenius term when the perturbation ansatz is inserted.

Substituting $T=T_{\infty }+\varepsilon \;\vartheta$ into the exponential term in Equation (5) and expanding about $T_{\infty }$ yields

$$\exp\;\left(-\frac{A_{r}}{T_{\infty }+\varepsilon \;\vartheta }\right)\approx \exp\;\left(-\frac{A_{r}}{T_{\infty }}\right)\exp\left(\vartheta \right),$$

so that the small parameter ε is eliminated from the argument of the exponential, and the temperature dependence appears in a leading-order form. This makes it possible to identify a consistent perturbation problem.

A remaining issue is the scaling of the source term. At first sight, the baseline reaction term appears as a zeroth-order contribution, while the base state $\left(T_{0}=T_{\infty }\right)$ has no spatial gradients and therefore cannot balance it through heat conduction. A steady near-critical balance can therefore only be achieved if the reaction term is effectively of order 𝒪(ε), so that it can be balanced by the 𝒪(ε) conductive contribution arising from the perturbation.

In practice, this corresponds to a consistent rescaling of the source term (formally multiplying by a factor ε·1/ε=1) so that reaction and conduction enter at the same asymptotic order, allowing a non-trivial first-order equation to be extracted.

This construction corresponds to the mathematical distinguished limit in high-activation-energy asymptotics [14].

Substituting the perturbation series into the decoupled energy equation and collecting the balanced 𝒪(ε) terms yields the first-order ordinary differential equation (ODE):

(A2) $$\frac{d^{2}\vartheta }{dx^{2}}+\left(\frac{\sigma_{1}}{1+\sigma_{1}x}+\frac{\sigma_{2}}{1+\sigma_{2}x}\right)\frac{d\vartheta }{dx}+\beta \exp\left(\vartheta \right)=0,$$

where the rescaled 𝒪(1) dimensionless stability parameter β is defined as:

(A3) $$\beta =Da\;C_{\alpha }\exp\left(-\frac{A_{r}}{T_{\infty }}\right)\frac{1}{\varepsilon }.$$

The factor 1/ε arises directly from the source-term rescaling introduced above, ensuring that reaction and conduction contributions enter at the same asymptotic order.

### Appendix A.3. Boundary Conditions

The dimensionless Robin conditions applied in the main text lead, at first order, to:

(A4) $$\frac{d\vartheta }{d\tilde{x}}-Bi_{d}\;\vartheta =0,\;\tilde{x}=-1,$$

(A5) $$\frac{d\vartheta }{d\tilde{x}}+Bi_{u}\;\vartheta =0,\;\tilde{x}=+1.$$

Equations (A2)–(A5) form a nonlinear boundary-value problem for ϑ(x) with β as a parameter.

### Appendix A.4. Definition of the Critical Stability Limit

A stationary solution exists only if the heat-generation term $\beta \;e^{\vartheta }$ can be balanced by conduction in the curved shell. The critical stability limit is therefore defined as the value $\beta_{\mathrm{crit}}$ where the solution to Equations (A2)–(A5) disappears. Because geometry and boundary conditions appear only in the differential operator and BCs, $\beta_{\mathrm{crit}}=\beta_{\mathrm{crit}}\left(\sigma_{1},\sigma_{2},Bi_{d},Bi_{u}\right)$.

### Appendix A.5. Peak Temperature Rise at Criticality

The first-order perturbation solution predicts a peak temperature rise at criticality of

(A6) $$\Delta T_{\mathrm{crit}}=\vartheta_{\max,\mathrm{crit}}\;\varepsilon =\vartheta_{\max,\mathrm{crit}}\;\frac{T_{\infty }^{2}}{A_{r}},$$

where $\vartheta_{\max,\mathrm{crit}}=\max_{x}\vartheta \left(x\right){|}_{\beta =\beta_{\mathrm{crit}}}$. By construction, $\vartheta_{\max,\mathrm{crit}}$ is an 𝒪(1) quantity, since the chosen scaling absorbs the strong temperature sensitivity of the Arrhenius term into the exponential, leaving only order-unity variations in the perturbation field.

## Appendix B. Numerical Methodology and Supplementary Code Structure

To ensure full reproducibility, the complete MATLAB code used in this study is provided as Supplementary Material. All quantitative results and figures presented in the manuscript were generated directly from these routines.

For the general reader, this appendix outlines the key numerical methods and computational workflow underlying the results. The implementation has been structured to allow efficient evaluation of individual cases (e.g., single parameter combinations or geometric configurations) without requiring full multi-parameter sweeps. Readers interested in detailed implementation aspects, including solver settings, convergence criteria, and function-level structure, are referred to the commented source code in the Supplementary Material.

The computational framework consists of two main components: (i) a boundary-value solver used to determine the critical stability parameter $\beta_{\mathrm{crit}}$, and (ii) a transient solver used to validate the perturbation-based predictions and determine critical Damköhler numbers.

### Appendix B.1. Primary Driver Routines

The main scripts coordinate the numerical solution procedures and parameter studies:

- Beta_crit_solver_invariants.m: This script solves the nonlinear boundary-value problem governing the perturbation temperature profile using MATLAB’s adaptive solver bvp4c. A continuation strategy in the parameter β is employed to identify the critical value $\beta_{\mathrm{crit}}$, defined as the upper limit for which a bounded steady solution exists under the specified boundary conditions.
  In addition to determining $\beta_{\mathrm{crit}}$, the script performs systematic parameter sweeps over curvature and Biot numbers to generate the data required for the semi-analytical stability model. These data are used to fit compact response surfaces for $\beta_{\mathrm{crit}}\left(\sigma_{1},\sigma_{2},Bi_{d},Bi_{u}\right)$ and to extract the coefficients appearing in the semi-analytical expressions presented in the main text.
  For the symmetric heating case ($Bi_{u}=Bi_{d}$), a dedicated sweep over Biot numbers is carried out to quantify the dependence of the offset coefficient $c_{0}$ on Bi.
  For the asymmetric heating configuration ($Bi_{u}=1000$, $Bi_{d}$ variable), the coefficients of the semi-analytical model are obtained by performing a sequence of curvature sweeps at fixed values of $Bi_{d}$. The resulting datasets are stored in CSV format and subsequently processed by the post-processing routine Plot_coeffs_asymmetric.m. This script performs the regression of the response-surface coefficients as functions of $Bi_{d}$ and generates the corresponding plots presented in the manuscript.
- Thermal_runaway_driver.m: This routine provides the main interface to the fully coupled transient thermo-kinetic solver and serves as the reference tool for generating ’exact’ numerical solutions. It is used extensively throughout the study to compute critical Damköhler numbers $Da_{\mathrm{crit}}$ and to validate the perturbation-based stability predictions obtained from the boundary-value formulation.
  At its core, the script employs a one-dimensional through-thickness solver for the coupled heat-transfer and cure-kinetics equations, together with a bracketing and root-finding procedure to classify solutions as stable or runaway based on their temperature evolution. The resulting thresholds are used as a benchmark for assessing the accuracy and range of validity of the semi-analytical model.
  The implementation is organized using a case-based control structure that enables a range of targeted analyses, including single-point simulations for rapid inspection of temperature profiles, systematic parameter sweeps over curvature and boundary conditions, and dedicated studies for model calibration, robustness assessment, and dynamical behaviour near the stability limit. This flexible structure allows both rapid exploratory calculations and more extensive validation studies within a unified computational framework.
- Laminate_Stability_3d_Landscape.m: This script implements the quasi-3D analysis used to assess the validity of the locally one-dimensional assumption. The geometry is discretized by first constructing an orthogonal grid on the mid-surface and then extending it through the thickness using the local principal curvatures, resulting in a structured three-dimensional mesh of frustum-like elements.
  For each surface location, the through-thickness temperature field is computed using the one-dimensional transient solver. These local solutions are assembled into a full three-dimensional temperature field, which is then used to evaluate heat fluxes across each element. Transverse fluxes (through the thickness) are obtained directly from the 1D solution combined with the corresponding face areas, while lateral (in-plane) fluxes are approximated using second-order finite differences applied to the spatially distributed temperature field.
  To ensure consistency, the adaptive time discretisations of the individual 1D solutions are synchronised onto a common temporal framework. The flux comparison is performed at the most critical instant, defined as the time at which the least stable element reaches its peak temperature. At this point, the dominant thermal overshoot is developed and the ratio provides a conservative measure of the relative importance of in-plane heat conduction.
  The resulting flux ratios are evaluated throughout the domain to quantify the validity of the one-dimensional approximation across the geometry.

### Appendix B.2. Core Computational Subroutines

The driver scripts rely on a set of modular subroutines implementing the underlying numerical models:

- curesimu_double_curved: Core transient solver for the coupled heat-transfer and cure-kinetics equations, formulated using a method-of-lines approach [16]. The spatial domain is discretised using a second-order finite-volume scheme [17], resulting in a system of coupled nonlinear ordinary differential equations in time.
  The resulting semi-discrete system is integrated using MATLAB’s stiff solver ode15s, which is based on variable-order, variable-step backward differentiation formulas. This method is well suited to the present problem due to the stiffness introduced by the strongly temperature-dependent reaction kinetics. An adaptive time-stepping strategy is employed to satisfy user-defined error tolerances.
  The spatial discretisation accuracy has been assessed using Richardson extrapolation for representative cases near the stability limit. For a typical configuration ($A_{r}\approx 30$, $T_{\infty }\approx 2$, flat laminate, Da close to critical), the estimated error in peak temperature is below $2\times 10^{-4}$. This level of accuracy is sufficient to reliably resolve both the stability threshold and the qualitative features of the solution relevant to the present study.
  Accurate evaluation of higher-order temperature derivatives is required for the classification of solution behaviour. Because numerical differentiation amplifies discretisation and solver noise, relatively strict solver tolerances are necessary to ensure smooth and physically consistent temperature profiles.
  In practice, the tolerances used in ode15s (relative tolerance $10^{-8}$, absolute tolerance $10^{-10}$) were found to provide sufficiently smooth solutions for robust classification of thermal behaviour. These settings were determined empirically by monitoring the convergence and smoothness of the temperature field and its derivatives.
- Tbispeaks: Post-processing routine used to classify transient solutions as stable or runaway based on the curvature of the temperature-time signal. The method is based on analysing the second temporal derivative of the temperature, which is computed using finite differences for non-uniform time discretisations.
  For super-critical cases, the second derivative typically exhibits three distinct peaks: an initial peak associated with heating from the initial condition, a second peak corresponding to the onset of exothermic acceleration, and a third peak related to the subsequent cooling phase. In contrast, sub-critical cases exhibit only the initial heating peak, followed by a monotonically decreasing temperature slope. This difference provides a robust basis for classification.
  To reliably isolate the relevant peak, the signal is filtered in time. Early-time data (typically t<0.2) are excluded to eliminate the initial heating peak. The temperature maximum is first identified, and the analysis window is truncated at this time to ensure that only the rising portion of the thermal response is considered, thereby avoiding spurious detection of peaks associated with the cooling phase.
  Peak detection is performed using MATLAB’s findpeaks function with a minimum peak prominence threshold (typically $10^{-4}$), which was determined empirically to provide robust detection across the parameter ranges considered.
- Find_Da_crit_double_curved: Automated routine for determining the critical Damköhler number $Da_{\mathrm{crit}}$ using the transient solver and the classification criterion described above.
  The method proceeds in two stages. First, a robust bracketing procedure is used to identify lower and upper bounds for $Da_{\mathrm{crit}}$. Starting from an initial guess, the algorithm evaluates the solution behaviour via the Tbispeaks criterion and systematically adjusts the Damköhler number until a strict bracket is obtained, i.e., a lower bound corresponding to stable behaviour and an upper bound corresponding to thermal runaway.
  Once a valid bracket has been established, the critical value is computed using a root-finding procedure (fzero), applied to the classification signal. The combination of strict signal filtering in Tbispeaks and the bracketing strategy effectively eliminates spurious roots associated with numerical artefacts in the classification signal, ensuring robust identification of $Da_{\mathrm{crit}}$ across the parameter ranges investigated.
  While the method has proven reliable in all tested cases, the modular structure of the implementation allows straightforward inspection of individual solutions if needed. In particular, the single-case diagnostic mode of the transient driver provides direct access to the temperature history, its second derivative, and the active filtering window, enabling verification of the classification in potentially ambiguous situations.

## Appendix C. Theoretical Range of Validity

The purpose of this appendix is to examine the internal consistency of the locally one-dimensional approximation used in the perturbation analysis. The procedure is an *a posteriori* self-consistency check: the perturbation solution derived in Section 2 under the assumption of negligible lateral conduction is used to estimate the lateral conductive term that was omitted. If this reconstructed lateral contribution is much smaller than the retained transverse conductive contribution, the assumed ordering of heat-flux terms is self-consistent. If not, the locally one-dimensional approximation would be contradicted by its own flux balance.

The analytical part of the appendix develops this check from a local control-volume balance. A small surface patch on the laminate mid-surface is extruded through the thickness, and the net lateral conductive divergence within this volume is compared with the corresponding transverse conductive contribution. This leads to the flux-ratio criterion

$$R_{\mathrm{flux}}=\left|\frac{{\bar{Q}}_{\mathrm{lat},\mathrm{net}}}{{\bar{Q}}_{\mathrm{trans}}}\right|,$$

where values $R_{\mathrm{flux}}\ll 1$ indicate that lateral conduction has only a minor influence on the local heat balance.

Because this analytical estimate is evaluated on the steady-state solution manifold generated by the perturbation theory, no synchronization of transient temperature histories is required. Different surface points are compared through their local steady solutions, parameterized by the local geometry and the corresponding value of $\beta_{\mathrm{crit}}$. The purpose is not to reconstruct a transient three-dimensional temperature field, but to test whether the neglected lateral term would significantly perturb the steady-state stability criterion itself.

As an independent consistency check, a quasi-three-dimensional numerical analysis is also performed. This calculation retains the local through-thickness thermal formulation, but evaluates lateral and transverse fluxes directly on an extruded shell geometry using transient temperature histories obtained from the fully coupled kinetic-rate and energy equations. Since these numerical solutions are time-dependent, synchronization is required in that case. The analytical and quasi-three-dimensional calculations therefore address the same physical question from complementary perspectives.

### Appendix C.1. Control-Volume Formulation of the Flux Ratio

We begin by setting up a small control volume (CV) at a general point (u,v,z) in the laminate with sides du, dv and dz (see Figure A1). To simplify the following discussion we will introduce a shorthand notation for the faces where east (*e*) and west (*w*) denote the high and low *u*-direction, north (*n*) and south (*s*) denote the high and low *v*-direction and top (*t*) and bottom (*b*) denote the high and low *z*-direction.

If we keep u,v,du and dv constant and vary *z* from −w to *w* a global control volume with a small footprint defined by du and dv is created. In physical space defined with a global Cartesian coordinate system the dimensions of the frustum-shaped control volume depend on the coordinate transformation from the Cartesian system to the body-fitted (u,v,z). Its lateral edge lengths will stretch or contract depending on the principal radii of curvature while the top and bottom edges will be circular arcs defined by the principal radii of the mid-surface and the value of the through-thickness coordinate *z*.

On the mid-surface at z=0, the physical line elements corresponding to coordinate increments du and dv are

$$ds_{u}=A\left(u,v\right)\;du,\;ds_{v}=B\left(u,v\right)\;dv,$$

Here *A* and *B* are obtained from the coordinate transformation between the global Cartesian position vector and the local orthogonal body-fitted surface coordinates. When the surface patch is displaced a distance *z* along the surface normal, these lengths are modified by the local principal curvatures. Thus, in the extruded shell coordinates,

$$h_{u}=A\left(1+z\kappa_{1}\right),\;h_{v}=B\left(1+z\kappa_{2}\right),\;h_{z}=1,$$

where $h_{u}$, $h_{v}$, and $h_{z}$ are the physical scale factors in the *u*, *v*, and *z* directions, respectively. The sign convention is chosen such that a positive curvature increases the corresponding physical line element when moving in the positive *z*-direction. Equivalently, using the dimensionless thickness coordinate x=z/w and $\sigma_{i}=w\kappa_{i}$,

$$h_{u}=A\left(1+x\sigma_{1}\right),\;h_{v}=B\left(1+x\sigma_{2}\right),\;dz=w\;dx.$$

To leading order for an infinitesimal cell, the areas of the faces normal to the *u*-direction are

$$dA_{u}=h_{v}\;dv\;dz.$$

The values on the east and west faces are obtained by evaluating this expression at the corresponding face locations. The corresponding face areas of the infinitesimal control volume are therefore

$$dA_{e}=dA_{w}=h_{v}\;dv\;dz=B\left(1+z\kappa_{2}\right)\;dv\;dz,$$

for the faces normal to the *u*-direction,

$$dA_{n}=dA_{s}=h_{u}\;du\;dz=A\left(1+z\kappa_{1}\right)\;du\;dz,$$

for the faces normal to the *v*-direction, and

$$dA_{t}=dA_{b}=h_{u}h_{v}\;du\;dv=AB\left(1+z\kappa_{1}\right)\left(1+z\kappa_{2}\right)\;du\;dv,$$

for the transverse faces normal to the *z*-direction. These expressions are simply the curvilinear counterpart of the Cartesian face areas dy·dz, dx·dz, and dx·dy.

The dimensional conductive heat-flux vector is

q=−Λ∇T.

For the present flux-ratio estimate, we distinguish between the in-plane conductivity $\lambda_{\|}$ and the through-thickness conductivity $\lambda_{⊥}$. The heat flow into the control volume through a face with outward unit normal $n_{f}$ and area $dA_{f}$ is

$$d{\dot{Q}}_{f}^{\mathrm{in}}=-q\cdot n_{f}\;dA_{f}=\lambda \;\nabla T\cdot n_{f}\;dA_{f},$$

where $\lambda =\lambda_{\|}$ for lateral faces and $\lambda =\lambda_{⊥}$ for transverse faces.

In an orthogonal coordinate system, the gradient components are scaled by the corresponding physical scale factors:

$$\nabla T=\frac{1}{h_{u}}\frac{\partial T}{\partial u}\;e_{u}+\frac{1}{h_{v}}\frac{\partial T}{\partial v}\;e_{v}+\frac{\partial T}{\partial z}\;e_{z}.$$

Because the coordinate system is orthogonal, the outward normals of the east–west, north–south, and top–bottom face pairs are aligned with

$$\pm e_{u},\;\pm e_{v},\;\pm e_{z},$$

respectively. The scalar product $\nabla T\cdot n_{f}$ therefore simply extracts the corresponding normal derivative on each face, with the sign determined by whether the face is on the positive or negative side of the control volume.

It is useful to define the following scaled conductive quantities:

$$F_{u}=\lambda_{\|}\frac{h_{v}}{h_{u}}\frac{\partial T}{\partial u},\;F_{v}=\lambda_{\|}\frac{h_{u}}{h_{v}}\frac{\partial T}{\partial v},$$

and

$$F_{z}=\lambda_{⊥}h_{u}h_{v}\frac{\partial T}{\partial z}.$$

With heat flow into the control volume taken as positive, the net contribution from the east and west faces is

$$d{\dot{Q}}_{ew}^{\mathrm{in}}=\left[F_{u}\left(u+du,v,z\right)-F_{u}\left(u,v,z\right)\right]\;dv\;dz.$$

A Taylor expansion about *u* gives

$$d{\dot{Q}}_{ew}^{\mathrm{in}}=\frac{\partial F_{u}}{\partial u}\;du\;dv\;dz+𝒪\left(du^{2}\;dv\;dz\right).$$

Similarly, the net contribution from the north and south faces is

$$d{\dot{Q}}_{ns}^{\mathrm{in}}=\left[F_{v}\left(u,v+dv,z\right)-F_{v}\left(u,v,z\right)\right]\;du\;dz,$$

and therefore

$$d{\dot{Q}}_{ns}^{\mathrm{in}}=\frac{\partial F_{v}}{\partial v}\;du\;dv\;dz+𝒪\left(dv^{2}\;du\;dz\right).$$

The corresponding transverse contribution from the top and bottom faces of a small cell extending from *z* to z+dz is

$$d{\dot{Q}}_{tb}^{\mathrm{in}}=\left[F_{z}\left(u,v,z+dz\right)-F_{z}\left(u,v,z\right)\right]\;du\;dv,$$

which gives

$$d{\dot{Q}}_{tb}^{\mathrm{in}}=\frac{\partial F_{z}}{\partial z}\;du\;dv\;dz+𝒪\left(dz^{2}\;du\;dv\right).$$

The Taylor expansions show that the net contributions from all three face pairs are proportional to the same coordinate footprint dudv. For the lateral faces this factor appears because the net warming or cooling is obtained from the difference between opposite faces, not from the flux through an individual face.

We now integrate these infinitesimal contributions through the thickness of the laminate. For the lateral faces, summing all slices from z=−w to z=w gives

(A7) $${\dot{Q}}_{\mathrm{lat},\mathrm{net}}^{\mathrm{in}}=du\;dv\int_{-w}^{w}\left(\frac{\partial F_{u}}{\partial u}+\frac{\partial F_{v}}{\partial v}\right)dz.$$

For the transverse contribution, the corresponding integration over all infinitesimal cells gives

(A8) $${\dot{Q}}_{\mathrm{trans}}^{\mathrm{in}}=du\;dv\int_{-w}^{w}\frac{\partial F_{z}}{\partial z}dz.$$

The integral in Equation (A8) simply represents the telescopic cancellation of all internal top–bottom fluxes. Only the transverse fluxes through the two outer laminate surfaces remain:

(A9) $${\dot{Q}}_{\mathrm{trans}}^{\mathrm{in}}=du\;dv\left[F_{z}\left(u,v,w\right)-F_{z}\left(u,v,-w\right)\right].$$

The signs depend on the convention used for inward and outward fluxes, but the final flux ratio is based on absolute values.

Equations (A7) and (A8) show explicitly that both the net lateral contribution and the transverse contribution contain the same footprint factor dudv. This factor is therefore not part of the physical validity criterion and cancels when the flux ratio is formed. Thus,

(A10) $$R_{\mathrm{flux}}=\left|\frac{\int_{-w}^{w}\left(\frac{\partial F_{u}}{\partial u}+\frac{\partial F_{v}}{\partial v}\right)dz}{\int_{-w}^{w}\frac{\partial F_{z}}{\partial z}dz}\right|.$$

Equation (A10) shows explicitly that the coordinate footprint dudv cancels in the ratio. The remaining difficulty is that the lateral operator still contains the *z*-dependent scale-factor ratios $h_{v}/h_{u}$ and $h_{u}/h_{v}$. Retaining these factors would lead to a weighted through-thickness integral of lateral surface operators on the parallel surfaces of the shell.

For the analytical estimate used here, we take the leading-order slender-shell approximation of this expression by evaluating the lateral metric factors on the mid-surface. Thus,

$$\frac{h_{v}}{h_{u}}=\frac{B}{A}\frac{1+z\kappa_{2}}{1+z\kappa_{1}}=\frac{B}{A}+𝒪\left(w\kappa \right),$$

and similarly

$$\frac{h_{u}}{h_{v}}=\frac{A}{B}+𝒪\left(w\kappa \right).$$

This approximation converts the exact weighted lateral operator into the Laplace–Beltrami operator on the mid-surface. The neglected terms are higher-order corrections in the thickness-to-curvature-radius ratio and primarily affect the through-thickness profile factor. They do not alter the leading geometry dependence that is used below to assess whether lateral conduction is small compared with transverse conduction.

With this leading-order reduction, the numerator of Equation (A10) becomes

(A11) $$\int_{-w}^{w}\left(\frac{\partial F_{u}}{\partial u}+\frac{\partial F_{v}}{\partial v}\right)dz\approx \lambda_{\|}AB\int_{-w}^{w}\nabla_{2D,\mathrm{phys}}^{2}T\;dz,$$

where

(A12) $$\nabla_{2D,\mathrm{phys}}^{2}T=\frac{1}{AB}\left[\frac{\partial }{\partial u}\left(\frac{B}{A}\frac{\partial T}{\partial u}\right)+\frac{\partial }{\partial v}\left(\frac{A}{B}\frac{\partial T}{\partial v}\right)\right]$$

is the physical Laplace–Beltrami operator on the laminate mid-surface. Although the metric coefficients *A* and *B* are evaluated on the mid-surface, the temperature field T(u,v,z) may still depend on the through-thickness coordinate. Thus, $\nabla_{2D,\mathrm{phys}}^{2}T$ should be interpreted as the surface Laplacian of the lateral temperature variation at fixed *z*.

The denominator of Equation (A10) is obtained directly from the transverse boundary contribution,

$$\int_{-w}^{w}\frac{\partial F_{z}}{\partial z}dz=F_{z}\left(u,v,w\right)-F_{z}\left(u,v,-w\right).$$

Using

$$F_{z}=\lambda_{⊥}h_{u}h_{v}\frac{\partial T}{\partial z},$$

this becomes

(A13) $$\int_{-w}^{w}\frac{\partial F_{z}}{\partial z}dz=\lambda_{⊥}AB\;B_{z}\left[T\right],$$

where the transverse boundary-gradient operator is defined as

(A14) $$\begin{array}{cc} B_{z}\left[T\right]= & \left(1+w\kappa_{1}\right)\left(1+w\kappa_{2}\right){\left.\frac{\partial T}{\partial z}\right|}_{z=w}\\ \; & -\left(1-w\kappa_{1}\right)\left(1-w\kappa_{2}\right){\left.\frac{\partial T}{\partial z}\right|}_{z=-w}. \end{array}$$

The signs follow from the convention used for the net inward heat flow. Since the final flux ratio is taken in absolute value, the sign convention does not affect the validity estimate.

Substituting Equations (A11) and (A13) into Equation (A10) gives the leading-order control-volume estimate

(A15) $$R_{\mathrm{flux}}\approx \left|\frac{\lambda_{\|}AB\int_{-w}^{w}\nabla_{2D,\mathrm{phys}}^{2}T\;dz}{\lambda_{⊥}AB\;B_{z}\left[T\right]}\right|=\frac{\lambda_{\|}}{\lambda_{⊥}}\left|\frac{\int_{-w}^{w}\nabla_{2D,\mathrm{phys}}^{2}T\;dz}{B_{z}\left[T\right]}\right|.$$

This result shows explicitly that both the coordinate footprint dudv and the physical mid-surface metric factor AB cancel in the ratio. The numerator measures the net lateral warming or cooling accumulated over the four lateral faces, while the denominator measures the net transverse heat extraction through the two outer laminate surfaces.

To connect Equation (A15) with the dimensionless perturbation formulation, we introduce x=z/w, scale the surface coordinates with *w*, and write the temperature perturbation in dimensionless form as ϑ. Then

$$\nabla_{2D,\mathrm{phys}}^{2}=w^{-2}\nabla_{2D}^{2},\;\frac{\partial }{\partial z}=w^{-1}\frac{\partial }{\partial x}.$$

The resulting factors of *w*, as well as the temperature scale, cancel between numerator and denominator. In the leading-order slender-shell approximation used above, the curvature-dependent transverse area factors in $B_{z}\left[T\right]$ are also replaced by unity. The dimensionless transverse denominator therefore reduces to

$$B_{z}\left[T\right]\approx \left[{\left.\frac{\partial \vartheta }{\partial x}\right|}_{x=1}-{\left.\frac{\partial \vartheta }{\partial x}\right|}_{x=-1}\right].$$

### Appendix C.2. Relating Surface Temperature to Local Stability

The control-volume result above shows that the lateral contribution is governed by the surface Laplacian of the temperature field. To evaluate this quantity analytically, we need a representation of how the local through-thickness temperature field varies from point to point over the shell surface. Since the present validity estimate is tied to the steady-state perturbation analysis, we consider the family of local one-dimensional steady solutions parameterised by the surface coordinates.

Close to the critical point, the dominant variation along this family is the amplitude of the temperature overshoot rather than the detailed shape of the through-thickness profile. This follows from the structure of the local steady boundary-value problem: transverse diffusion strongly constrains the admissible temperature shapes, while the approach to the fold is primarily expressed as growth of the leading thermal mode. We therefore write the local perturbation field in the separated form

(A16) $$\vartheta \left(x,u,v\right)\approx \vartheta_{\mathrm{peak}}\left(u,v\right)f\left(x\right),$$

where f(x) is a smooth normalized through-thickness profile satisfying maxf=1, and $\vartheta_{\mathrm{peak}}\left(u,v\right)$ carries the spatial variation over the mid-surface.

This approximation should be viewed as a leading-mode representation of the local steady solution branch rather than as an assumption that all exact BVP profiles are identical. If desired, f(x) could be replaced by the numerically computed critical profile from the local boundary-value problem. In the present flux-ratio estimate, however, the profile shape enters only through a thickness-integrated factor of order unity, whereas the dominant spatial variation is contained in $\vartheta_{\mathrm{peak}}\left(u,v\right)$. The approximation is therefore sufficient for estimating whether lateral conduction remains small compared with transverse conduction.

To relate $\vartheta_{\mathrm{peak}}$ to the local geometry, we use the fact that the critical Damköhler number varies over the surface through the stability factor

$$Da_{\mathrm{crit}}\left(u,v\right)=Da_{\mathrm{crit},\mathrm{flat}}\;\beta_{\mathrm{crit}}\left(\sigma_{1},\sigma_{2}\right).$$

For a given manufacturing cycle, the process Damköhler number Da is spatially uniform because the nominal half-thickness, material properties, and external temperature scale are fixed. The local proximity to the steady runaway limit therefore varies primarily through $\beta_{\mathrm{crit}}$.

Near the critical branch, the temperature overshoot scales with this proximity to the local stability limit (see Figure 6). This gives the leading-order relation

(A17) $$\vartheta_{\mathrm{peak}}\left(u,v\right)\approx \vartheta_{\mathrm{peak},\mathrm{crit}}\frac{Da}{Da_{\mathrm{crit},\mathrm{flat}}\;\beta_{\mathrm{crit}}\left(\sigma_{1},\sigma_{2}\right)}=\frac{G}{\beta_{\mathrm{crit}}\left(\sigma_{1},\sigma_{2}\right)},$$

where

$$G=\vartheta_{\mathrm{peak},\mathrm{crit}}\frac{Da}{Da_{\mathrm{crit},\mathrm{flat}}}$$

is spatially constant for the manufacturing cycle considered.

Thus, the perturbation analysis converts the difficult question of lateral temperature variation into a geometrical one: regions with lower local stability, i.e., smaller $\beta_{\mathrm{crit}}$, develop larger steady temperature overshoots and therefore stronger lateral gradients.

### Appendix C.3. Definition of the Flux-Ratio Criterion

The control-volume formulation shows that the relevant lateral contribution is the net lateral conductive divergence, not the absolute flux through an individual lateral face. To compare this term with the retained through-thickness conduction term, we integrate the dimensionless energy balance over the control volume.

Using the separated representation

$$\vartheta \left(x,u,v\right)\approx \vartheta_{\mathrm{peak}}\left(u,v\right)f\left(x\right),$$

the transverse conductive contribution becomes

(A18) $${\bar{Q}}_{\mathrm{trans}}=\int_{-1}^{1}\frac{\partial^{2}\vartheta }{\partial x^{2}}\;dx=\vartheta_{\mathrm{peak}}\left[f^{\prime }\left(1\right)-f^{\prime }\left(-1\right)\right].$$

Similarly, the net lateral conductive contribution is

(A19) $${\bar{Q}}_{\mathrm{lat},\mathrm{net}}=\int_{-1}^{1}\Xi_{k}\nabla_{2D}^{2}\vartheta \;dx=\Xi_{k}\left(\nabla_{2D}^{2}\vartheta_{\mathrm{peak}}\right)\int_{-1}^{1}f\left(x\right)\;dx,$$

where $\Xi_{k}=\lambda_{\|}/\lambda_{⊥}$. For isotropic conductivity, $\Xi_{k}=1$.

The flux-ratio criterion is then defined as

(A20) $$R_{\mathrm{flux}}=∥\frac{{\bar{Q}}_{\mathrm{lat},\mathrm{net}}}{{\bar{Q}}_{\mathrm{trans}}}∥=\Xi_{k}\underset{\mathrm{surface}-\mathrm{geometry}\;\mathrm{term}}{\underset{︸}{∥\frac{\nabla_{2D}^{2}\vartheta_{\mathrm{peak}}}{\vartheta_{\mathrm{peak}}}∥}}\;\underset{\mathrm{profile}-\mathrm{shape}\;\mathrm{term}\;𝒫}{\underset{︸}{∥\frac{\int_{-1}^{1}f\left(x\right)\;dx}{f^{\prime }\left(1\right)-f^{\prime }\left(-1\right)}∥}}.$$

This expression is useful because it separates the problem into three physically distinct factors. The factor $\Xi_{k}$ accounts for possible thermal-conductivity anisotropy. The profile-shape term 𝒫 depends only on the normalized through-thickness temperature distribution. The remaining factor contains the spatial variation of the peak temperature over the shell surface and is therefore the part controlled by geometry.

For smooth through-thickness profiles, 𝒫 is a quantity of order unity. For example, the classical parabolic profile $f\left(x\right)=1-x^{2}$ gives

$$𝒫=\frac{1}{3},$$

whereas the lowest diffusion mode f(x)=cos(πx/2) gives

$$𝒫=\frac{4}{\pi^{2}}\approx 0.405.$$

Thus, for the smooth profiles relevant here, the dominant spatial variation in $R_{\mathrm{flux}}$ originates from the surface-geometry term.

Substituting the inverse relation

$$\vartheta_{\mathrm{peak}}=\frac{G}{\beta_{\mathrm{crit}}\left(\sigma_{1},\sigma_{2}\right)}$$

into the geometry term eliminates the global process constant G. Applying the quotient rule gives

(A21) $$\frac{\nabla_{2D}^{2}\vartheta_{\mathrm{peak}}}{\vartheta_{\mathrm{peak}}}=-\frac{\nabla_{2D}^{2}\beta_{\mathrm{crit}}}{\beta_{\mathrm{crit}}}+2\frac{{∥\nabla_{2D}\beta_{\mathrm{crit}}∥}^{2}}{\beta_{\mathrm{crit}}^{2}}.$$

Equation (A21) is the central result of the analytical flux-ratio estimate. It shows that the process intensity cancels from the lateral-to-transverse flux ratio: the remaining variation is governed by the spatial gradients and Laplacian of the local stability function $\beta_{\mathrm{crit}}\left(\sigma_{1},\sigma_{2}\right)$. In other words, lateral conduction becomes important not simply because the laminate is curved, but because the local stability limit varies over the surface. This is the key simplification that makes a geometry-based validity criterion possible.

### Appendix C.4. Evaluation for Representative Test Geometries

The analytical flux-ratio expression derived above is local: it can be evaluated at any point on a smooth laminate mid-surface for which the curvatures and their spatial derivatives are known. For a complete component, the relevant engineering quantity is therefore the maximum value of the flux ratio over the surface. In the following, this point is referred to as the “worst point”. It should be emphasized that this does not necessarily coincide with the point of largest curvature magnitude. According to Equation (A21), the flux ratio is controlled by the gradients and Laplacian of the stability function $\beta_{\mathrm{crit}}\left(\sigma_{1},\sigma_{2}\right)$, and therefore by how rapidly the local stability limit varies over the surface.

For the representative geometries considered here, symmetry makes the worst-point evaluation particularly simple. At the relevant symmetry points of the saddle and torus, the first spatial derivatives of the dimensionless curvatures vanish,

$$\nabla_{2D}\sigma_{1}=\nabla_{2D}\sigma_{2}=0.$$

Consequently, the gradient-square contribution in Equation (A21) vanishes locally, and the geometry term reduces to

$$\frac{\nabla_{2D}^{2}\vartheta_{\mathrm{peak}}}{\vartheta_{\mathrm{peak}}}=-\frac{\nabla_{2D}^{2}\beta_{\mathrm{crit}}}{\beta_{\mathrm{crit}}}.$$

This simplification is used only for the tabulated test cases below. For a general surface, the full expression in Equation (A21), including the gradient-square term, should be evaluated.

Since

$$\beta_{\mathrm{crit}}=\beta_{\mathrm{crit}}\left(\sigma_{1},\sigma_{2}\right),$$

the Laplacian of the stability function can be expanded by the chain rule. At the symmetry points considered here, where the curvature gradients vanish, this gives

$$\nabla_{2D}^{2}\beta_{\mathrm{crit}}=\beta_{,1}\nabla_{2D}^{2}\sigma_{1}+\beta_{,2}\nabla_{2D}^{2}\sigma_{2},$$

where

$$\beta_{,i}=\frac{\partial \beta_{\mathrm{crit}}}{\partial \sigma_{i}}.$$

For the semi-analytical representation of the stability surface used in this work, both the symmetric and asymmetric boundary-condition cases can be written as

$$\beta_{\mathrm{crit}}=c_{0}+LS+a_{\beta }S^{2}+b_{\beta }P,$$

with

$$S=\sigma_{1}+\sigma_{2},\;P=\sigma_{1}\sigma_{2}.$$

The corresponding sensitivities are

$$\beta_{,1}=L+2a_{\beta }S+b_{\beta }\sigma_{2},$$

and

$$\beta_{,2}=L+2a_{\beta }S+b_{\beta }\sigma_{1}.$$

The constant offset $c_{0}$ does not enter these sensitivity derivatives, but it is retained when the normalized ratio

$$G_{\beta }=\left|\frac{\nabla_{2D}^{2}\beta_{\mathrm{crit}}}{\beta_{\mathrm{crit}}}\right|$$

is evaluated.

The final local flux-ratio estimate at the symmetry point is therefore

$$R_{\mathrm{flux}}=\Xi_{k}𝒫\left|\frac{\beta_{,1}\nabla_{2D}^{2}\sigma_{1}+\beta_{,2}\nabla_{2D}^{2}\sigma_{2}}{\beta_{\mathrm{crit}}}\right|,$$

where

$$\Xi_{k}=\frac{\lambda_{\|}}{\lambda_{⊥}}$$

is the conductivity anisotropy ratio and 𝒫 is the through-thickness profile factor. For the isotropic calculations considered below, $\Xi_{k}=1$. Since 𝒫<1 for the smooth through-thickness profiles considered here, the geometry factor $G_{\beta }$ provides a conservative upper estimate of the flux ratio.

The test geometries are chosen to probe distinct regions of curvature space. The straight cylinder represents the important limiting case of finite but spatially constant curvature. The symmetric saddle probes the anticlastic line $\sigma_{1}=-\sigma_{2}$, while the torus probes trajectories with one positive curvature component and one varying curvature component that will generate both synclastic and anticlastic curvature on the outer and inner diameter of the torus respectively. Together, these cases provide a compact but informative test of the geometry-based flux-ratio criterion.

For a straight cylinder with uniform thermal boundary conditions, both dimensionless curvatures are spatially constant. Hence

$$\nabla_{2D}\sigma_{i}=0,\;\nabla_{2D}^{2}\sigma_{i}=0,$$

and the analytical flux ratio vanishes identically. This is a particularly important result because cylindrical shells are common composite components. Within the assumptions of the present analysis, finite cylindrical curvature alone does not create net lateral conductive divergence; the locally one-dimensional runaway criterion is therefore valid for a straight cylinder with uniform boundary conditions, independently of wall thickness.

For a symmetric saddle described locally by

$$z\left(u,v\right)=\frac{u^{2}}{2a}-\frac{v^{2}}{2a},$$

the symmetry point is the saddle centre. The dimensionless curvatures are

$$\sigma_{1}=\frac{w}{a},\;\sigma_{2}=-\frac{w}{a},$$

and the corresponding dimensionless surface Laplacians are

$$\nabla_{2D}^{2}\sigma_{1}=-4{\left(\frac{w}{a}\right)}^{3},\;\nabla_{2D}^{2}\sigma_{2}=+4{\left(\frac{w}{a}\right)}^{3}.$$

For a torus with major radius *R* and minor radius *r*, the evaluated point is the inner equator, where the anticlastic character is strongest. With the sign convention used here,

$$\kappa_{1}=\frac{1}{r},\;\kappa_{2}=-\frac{1}{R-r}.$$

Thus,

$$\sigma_{1}=\frac{w}{r},\;\sigma_{2}=-\frac{w}{R-r}.$$

The poloidal curvature is constant on the torus, giving

$$\nabla_{2D}^{2}\sigma_{1}=0,$$

whereas the azimuthal curvature gives

$$\nabla_{2D}^{2}\sigma_{2}=\frac{w^{3}R}{r^{2}{\left(R-r\right)}^{2}}.$$

Table A1 lists the numerical quantities entering the geometry factor $G_{\beta }$ for the representative saddle and toroidal cases. The torus cases use R=1.0 and r=0.3, while the saddle case uses a=0.5. The stability-surface coefficients are those for the symmetric high-Biot-number case considered here,

$$c_{0}=0.88,\;L=0,\;a_{\beta }=0.121,\;b_{\beta }=0.427.$$

The tabulated value of $G_{\beta }$ is a conservative geometry estimate. The actual analytical flux-ratio estimate is obtained by multiplying $G_{\beta }$ by $\Xi_{k}𝒫$.

**Table A1:** Numerical quantities entering the symmetry-point geometry estimate $G_{\beta }$ for the representative saddle and toroidal test geometries. Here *w* is the laminate half-thickness. The torus cases use r=0.3 and R=1.0, with total thicknesses h=2w. The saddle case uses a=0.5 and w=0.1. The stability fit uses $c_{0}=0.88$, L=0, $a_{\beta }=0.121$, and $b_{\beta }=0.427$. The full analytical flux-ratio estimate is obtained as $R_{\mathrm{flux}}=\Xi_{k}𝒫G_{\beta }$.

Quantity	Saddle	Torus	Torus	Torus
h=2w	0.2	0.1	0.2	0.4
*w*	0.1	0.05	0.10	0.20
$\sigma_{1}$	0.2000	0.1667	0.3333	0.6667
$\sigma_{2}$	−0.2000	−0.0714	−0.1429	−0.2857
$\nabla_{2D}^{2}\sigma_{1}$	−0.0320	0	0	0
$\nabla_{2D}^{2}\sigma_{2}$	0.0320	0.00283	0.0227	0.1814
$S=\sigma_{1}+\sigma_{2}$	0	0.0952	0.1905	0.3810
$P=\sigma_{1}\sigma_{2}$	−0.0400	−0.0119	−0.0476	−0.1905
$\beta_{,1}$	−0.0854	−0.00745	−0.0149	−0.0298
$\beta_{,2}$	0.0854	0.0942	0.1884	0.3769
$\beta_{\mathrm{crit}}$	0.8629	0.8760	0.8641	0.8162
$\nabla_{2D}^{2}\beta_{\mathrm{crit}}$	0.00547	0.000267	0.00427	0.0684
$\left|\nabla_{2D}^{2}\beta_{\mathrm{crit}}/\beta_{\mathrm{crit}}\right|$	0.00633	0.000305	0.00494	0.0838

The final row of Table A1 shows that the conservative geometry factor remains small for the representative test cases. The saddle case gives $G_{\beta }=6.3\times 10^{-3}$, while the two thinner toroidal cases remain below $5\times 10^{-3}$. Even for the most severe toroidal case considered, corresponding to w=0.2 and h=0.4, the conservative geometry factor remains below 0.1. Since the actual flux ratio is obtained by multiplying this value by the profile factor 𝒫<1, the predicted lateral contribution remains only a few percent even in this deliberately severe case.

These estimates support the central interpretation of the analytical criterion. Curvature magnitude alone does not determine the importance of lateral conduction. A straight cylinder has finite curvature but zero net lateral conductive divergence under uniform thermal boundary conditions. The saddle and torus introduce spatially varying curvature and therefore non-zero gradients and Laplacians of $\beta_{\mathrm{crit}}$, but the resulting geometry factors remain small over the investigated range. The analytical estimate therefore suggests that the locally one-dimensional approximation is robust for smooth shell geometries unless the stability function varies rapidly over the surface.

### Appendix C.5. Scaling of the Flux Ratio with Curvature

The test-surface calculations above provide explicit flux-ratio estimates for a small set of representative geometries. It is also useful to understand why the geometry factors become small so rapidly for slender laminates on smooth tooling surfaces. For this purpose, we use a local Monge-patch argument. A Monge patch represents a smooth surface locally as a height function above its tangent plane. This viewpoint is closely connected to CAD geometry: although engineering surfaces are usually represented by spline-based descriptions such as Non-Uniform Rational B-Splines (NURBS) rather than by Taylor polynomials, the local curvature and curvature gradients are still obtained from derivatives of the surface representation. Thus, in a sufficiently small neighbourhood of any regular point, the mid-surface can be expanded in local coordinates aligned with the principal curvature directions. The quadratic terms describe the local principal curvatures, while the higher-order terms describe how those curvatures vary along the surface.

This analysis should not be interpreted as replacing the full flux-ratio criterion by a criterion based only on the principal radii of curvature. The exact analytical criterion remains Equation (A21), which depends on the surface gradients and Laplacian of $\beta_{\mathrm{crit}}\left(\sigma_{1},\sigma_{2}\right)$. The role of the Monge-patch analysis is instead to estimate how these derivatives scale for a smooth surface whose curvature varies over a characteristic geometric length scale.

Let the local surface be represented as

(A22) $$z\left(u,v\right)=\frac{u^{2}}{2R_{1}}+\frac{v^{2}}{2R_{2}}+\frac{C_{30}}{3!}u^{3}+\frac{C_{21}}{2!}u^{2}v+\frac{C_{12}}{2!}uv^{2}+\frac{C_{03}}{3!}v^{3}+𝒪\left(u^{4},v^{4}\right),$$

where $R_{1}$ and $R_{2}$ are the local principal radii of curvature. The quadratic terms determine the curvatures at the expansion point, whereas the higher-order terms describe how those curvatures change as one moves along the surface. Thus, curvature gradients are not discarded in this representation; they enter through the cubic and higher-order coefficients.

For a smooth manufacturable surface, it is natural to assume that both the curvature and its spatial variation are controlled by a characteristic geometric length scale *R*. This length scale should be understood as the length over which the curvature field changes appreciably, not merely as the smallest principal radius at the point. Under this smoothness assumption, dimensional consistency gives

$$\kappa_{i}∼R^{-1},\;\nabla_{\mathrm{phys}}\kappa_{i}∼R^{-2},\;\nabla_{\mathrm{phys}}^{2}\kappa_{i}∼R^{-3}.$$

Equivalently, in terms of the dimensionless curvatures

$$\sigma_{i}=w\kappa_{i},$$

and the dimensionless surface coordinates scaled with the laminate half-thickness *w*, one obtains

(A23) $$\sigma_{i}∼\epsilon ,\;{∥\nabla_{2D}\sigma_{i}∥}^{2}∼\epsilon^{4},\;\nabla_{2D}^{2}\sigma_{i}∼\epsilon^{3},$$

where

$$\epsilon =\frac{w}{R}.$$

Here *R* should be interpreted as the characteristic length scale over which the curvature field varies, rather than merely as the smallest local radius of curvature. The important point is that the Laplacian of the curvature field enters one order lower than the squared curvature-gradient term. This is why the leading correction associated with spatially varying curvature is expected to scale at least as $\epsilon^{3}$ for smooth surfaces.

Substitution of these estimates into the geometry term,

$$\frac{\nabla_{2D}^{2}\vartheta_{\mathrm{peak}}}{\vartheta_{\mathrm{peak}}}=-\frac{\nabla_{2D}^{2}\beta_{\mathrm{crit}}}{\beta_{\mathrm{crit}}}+2\frac{∥\nabla_{2D}\beta_{\mathrm{crit}}{∥}^{2}}{\beta_{\mathrm{crit}}^{2}},$$

gives the order-of-magnitude estimate

(A24) $$R_{\mathrm{flux}}∼\Xi_{k}𝒫\;𝒪\left(1\right)\left[\frac{\epsilon^{3}+\epsilon^{4}}{\beta_{\mathrm{crit}}}+\frac{\epsilon^{4}}{\beta_{\mathrm{crit}}^{2}}\right].$$

Here the terms proportional to $\epsilon^{3}$ and $\epsilon^{4}$ arise from the Laplacian of $\beta_{\mathrm{crit}}$, while the term proportional to $\epsilon^{4}$ arises from the squared-gradient contribution. The prefactor 𝒪(1) contains the local sensitivities of $\beta_{\mathrm{crit}}$ with respect to $\sigma_{1}$ and $\sigma_{2}$, and therefore depends on where the surface lies in curvature space.

Equation (A24) explains why lateral conduction is strongly suppressed for slender laminates on smooth tooling surfaces. As w/R decreases, the geometric derivatives that drive the lateral flux vanish rapidly. However, the estimate should be read as a scaling law, not as a universal bound. Geometries in which the curvature changes over a much shorter length scale than the local radius, or regions where $\beta_{\mathrm{crit}}$ varies rapidly in curvature space, must still be assessed using the full flux-ratio criterion in Equation (A21).

### Appendix C.6. Comparison with Quasi-Three-Dimensional Simulations

The analytical flux-ratio estimate developed above is based on the steady-state perturbation framework. As an independent consistency check, we also evaluate the same basic quantity numerically using a quasi-three-dimensional representation of the shell geometry. The purpose of this calculation is not to replace the analytical criterion, but to test whether the neglected lateral conductive contribution remains small when the temperature field is obtained from the transient, fully coupled kinetic-rate and energy equations.

#### Appendix C.6.1. Numerical Evaluation of the Flux Ratio

The quasi-three-dimensional model starts from a structured quadrilateral mesh on the laminate mid-surface. This surface mesh is extruded stepwise along the local surface normals to generate a three-dimensional shell mesh. Each surface cell therefore defines a curved thermal control volume extending through the thickness. For the slender laminates considered here, the thickness is small compared with the local radii of curvature, so the normal extrusion remains well defined and neighbouring cells form a conforming volume mesh.

Each extruded control volume is subdivided through the thickness into the same number of layers as used in the one-dimensional cure solver. The transient through-thickness heat-conduction problem, including reaction heat generation and the local curvature-dependent terms, is then solved for each thermal element using the fully coupled kinetic-rate and energy equations. This preserves the local through-thickness formulation while allowing lateral and transverse fluxes to be evaluated directly from the resulting three-dimensional arrangement of neighbouring elements.

Because the numerical solutions are transient, different surface locations may reach their largest temperature overshoots at different times. In contrast to the analytical steady-state estimate, a synchronization step is therefore required. The temperature histories are interpolated onto a common reference time, chosen as the time at which the hottest thermal element reaches its peak temperature. Lateral temperature gradients are then evaluated from neighbouring surface elements, while transverse gradients at the upper and lower surfaces are computed from the through-thickness temperature profiles. Combining these gradients with the geometrical face areas of the extruded mesh gives a direct numerical estimate of the local flux ratio for each thermal element.

The mesh generation and finite-volume flux evaluation follow the implementation described in the Supplementary MATLAB code.

#### Appendix C.6.2. Representative Test Geometries

The numerical study uses the same classes of test geometries as the analytical evaluation: a symmetric saddle surface and a toroidal pipe bend. These geometries were selected because they provide complementary, controlled probes of the flux-ratio response in curvature space. The saddle follows the anticlastic line

$$\sigma_{1}=-\sigma_{2},$$

whereas the toroidal geometries follow line scans with finite positive $\sigma_{1}$ and varying $\sigma_{2}$. Increasing the laminate thickness increases the dimensionless curvatures and therefore extends the range probed by each toroidal scan.

These test geometries should not be interpreted as universal bounds for arbitrary shells. Rather, they are representative probes of curvature-space regions that are expected to be relevant for practical composite structures. The saddle tests the strongly anticlastic direction, while the torus tests the effect of combining a positive curvature component with a second curvature component that varies from weakly positive or near-cylindrical states into anticlastic states.

#### Appendix C.6.3. Numerical Results

The analytical framework predicts three qualitative trends. First, the flux ratio should decrease rapidly as the thickness-to-radius ratio decreases. Second, regions with smoothly varying synclastic or near-cylindrical curvature should produce weak lateral-conduction corrections. Third, the largest deviations from the locally one-dimensional approximation should occur in anticlastic regions, where the stability function $\beta_{\mathrm{crit}}$ varies most rapidly.

Figure A2 summarizes the quasi-three-dimensional results. Panels (a)–(c) illustrate the procedure for the thickest toroidal laminate considered: the extruded shell geometry, the surface distribution of the computed flux ratio, and the through-thickness temperature profile in the hottest element at its peak time. These panels show directly that the large through-thickness gradients responsible for transverse heat extraction coexist with much smaller lateral conductive contributions.

The main quantitative comparison is shown in panel (d), where the computed flux ratio is plotted against $\sigma_{2}$ for the saddle and toroidal scans described above. Because the datasets correspond to different paths through the $\left(\sigma_{1},\sigma_{2}\right)$-plane, panel (d) should be interpreted as a set of controlled probes through the underlying flux-ratio surface, rather than as a single universal curve.

The numerical results support the analytical interpretation. Over the range

$$-0.2≲\sigma_{2}≲0.15,$$

the computed flux ratios remain below approximately

$$4\times 10^{-3}.$$

Thus, throughout most of the investigated curvature domain, lateral heat transport is negligible compared with transverse heat extraction. For more negative values of $\sigma_{2}$, corresponding to increasingly anticlastic regions, the flux ratio rises more rapidly and reaches the one-percent level at approximately

$$\sigma_{2}\approx -0.25.$$

This behaviour is consistent with the analytical criterion. Anticlastic regions have lower local stability margins under the symmetric heating conditions considered here. They therefore develop larger local temperature overshoots and stronger lateral temperature gradients, which in turn increase the lateral conductive heat flow. The quasi-three-dimensional simulations thus identify the same limiting region of curvature space as the analytical flux-ratio estimate.

The toroidal simulations provide an additional useful observation. Near cylindrical states, where $\sigma_{2}\approx 0$, increasing the positive curvature component $\sigma_{1}$ raises the flux-ratio level, but does not by itself cause a breakdown of the locally one-dimensional approximation. Over the directly probed range

$$0.167\leq \sigma_{1}\leq 0.667,$$

the flux ratio near $\sigma_{2}\approx 0$ remains below about 0.5%, even for the largest investigated value of $\sigma_{1}$. Positive curvature in the synclastic or near-cylindrical direction is therefore comparatively benign for the symmetric thermal boundary conditions considered here.

A useful quantitative comparison can be made by evaluating the analytical geometry estimate at curvature levels corresponding to the quasi-three-dimensional simulations. For the toroidal cases with

$$\sigma_{2}=-0.143\;\mathrm{and}\;\sigma_{2}=-0.286,$$

Table A1 gives

$$G_{\beta }=0.0049\;\mathrm{and}\;G_{\beta }=0.084,$$

respectively. Using a representative through-thickness profile factor of order 𝒫≈0.5, and $\Xi_{k}=1$, gives analytical flux-ratio estimates of approximately

$$2.5\times 10^{-3}\;\mathrm{and}\;4.2\times 10^{-2}.$$

Interpolation of the quasi-three-dimensional data at the same curvature levels gives corresponding values of approximately

$$1.0\times 10^{-3}\;\mathrm{and}\;1.5\times 10^{-2}.$$

The agreement is within roughly a factor of three. This level of agreement is satisfactory given that the analytical values are conservative symmetry-point estimates, whereas the quasi-three-dimensional values are extracted from finite surface scans of transient simulations. More importantly, the two approaches identify the same anticlastic region as limiting and predict flux ratios of the same order of magnitude.

Taken together, the quasi-three-dimensional simulations support the analytical flux-ratio analysis. The lateral contribution remains well below one percent over most of the investigated curvature range, rises most rapidly in anticlastic regions, and remains small near cylindrical states even for relatively large positive $\sigma_{1}$. These trends are precisely those expected from Equation (A21), where the flux ratio is governed by spatial variations of $\beta_{\mathrm{crit}}$ rather than by curvature magnitude alone.

The numerical results should not be interpreted as a universal proof for arbitrary shell geometries. They do, however, provide an important independent consistency check. The analytical estimate is based on the steady-state perturbation framework, whereas the quasi-three-dimensional calculation uses synchronized transient temperature fields from the fully coupled kinetic-rate and energy equations. The fact that both approaches predict small flux ratios for the representative test surfaces supports the use of the locally one-dimensional approximation for smooth shell geometries within the investigated curvature range.
